# Molecular mechanisms and structure—activity relationships of natural polysaccharides in ameliorating type 2 diabetes mellitus: a comprehensive review

**DOI:** 10.3389/fnut.2026.1810613

**Published:** 2026-05-29

**Authors:** Ying Zhou, Ruodi Yang, Qingfeng Wang, Jiaxin Li, Yufeng Yang, Yan Shi, Juntong Liu

**Affiliations:** 1 First Clinical College, Liaoning University of Traditional Chinese Medicine, Shenyang, China; 2 School of Basic Medicine, Liaoning University of Traditional Chinese Medicine, Shenyang, China; 3 Teaching and Experiment Center, Liaoning University of Traditional Chinese Medicine, Shenyang, China

**Keywords:** glucose metabolism, insulin resistance, molecular mechanisms, natural polysaccharides, pancreatic β-cells, structure–activity relationships, type 2 diabetes mellitus

## Abstract

Type 2 diabetes mellitus (T2DM) is a global metabolic pandemic affecting hundreds of millions of people, with current pharmacological therapies limited by adverse effects, long-term tolerability issues, and cost barriers. Natural polysaccharides—high-molecular-weight carbohydrate polymers derived from plants, fungi, marine organisms, and animal sources—have emerged as a promising class of multi-target bioactive agents for T2DM management. This comprehensive review first outlines the key pathophysiological mechanisms of T2DM, encompassing insulin resistance, pancreatic β-cell dysfunction, chronic inflammation, oxidative stress, and gut microbiota dysbiosis. We then systematically review the natural sources and structural classification of polysaccharides, alongside their extraction and purification methods. The core of this review examines the molecular mechanisms by which natural polysaccharides ameliorate T2DM: (1) enhancing insulin sensitivity and glucose metabolism via the PI3K/Akt and AMPK signaling pathways; (2) protecting pancreatic β-cells from apoptosis and promoting insulin secretion; (3) suppressing chronic inflammation through NF-κB and NLRP3 pathway inhibition; (4) attenuating oxidative stress via Nrf2/HO-1 pathway activation; and (5) restoring gut microbiota homeostasis, reinforcing intestinal barrier integrity, and elevating short-chain fatty acids production. Structure–activity relationship analyses indicate that hypoglycemic efficacy is tightly correlated with molecular weight, monosaccharide composition, glycosidic linkage types, degree of branching, three-dimensional conformation, and chemical derivatization. Finally, challenges surrounding clinical translation, standardization, and bioavailability are discussed, along with future research directions. This review provides a theoretical framework for the application of natural polysaccharides as functional foods, nutraceuticals, or lead compounds in T2DM prevention and treatment.

## 1 Introduction

Type 2 diabetes mellitus (T2DM) is one of the greatest public health problems faced by contemporary society. In 2021, the number of people with diabetes was as high as 529 million worldwide, and it is expected to exceed 1.31 billion by 2050. T2DM accounts for almost 96% of all such cases, while global health care costs due to diabetes reached as high as $966 billion in 2021 (1, 2). The disease predisposes to many debilitating clinical complications such as cardiovascular diseases, nephropathy, and retinopathy that exert profound adverse impacts on health and life of these patients (3, 4), as well as their mortality being higher than that for people who do not suffer from diabetes (5).

The main pathophysiology of T2DM is insulin resistance (IR), combined with gradual deterioration of β-cells in the pancreas (4). The traditional drugs used to treat T2DM are metformin in conjunction with new drugs such as sodium-glucose cotransporter-2 (SGLT2) inhibitors or glucagon-like peptide-1 (GLP-1) receptor agonists. While they have made significant progress in controlling blood sugar levels, these drugs are burdened with serious drawbacks (6). While large systematic network meta-analyses show that both SGLT2 inhibitors and GLP-1RA offer significant benefits in terms of CV and renal morbidity and mortality, they have serious adverse effects. In particular, the use of SGLT2 inhibitors could induce genital infections, while the administration of GLP-1 RAs might cause serious gastrointestinal side effects, and basal insulin plus thiazolidinediones are often associated with significant weight gain (7). The cost of chronic pharmacotherapy and poor drug compliance further limit their effectiveness. Therefore, there is a great need to develop new anti-diabetic drugs that are both effective and have low side effects.

Natural polysaccharides are natural high-molecular-weight carbohydrate macromolecules found ubiquitously in plants, fungi, marine organisms, and animals, and have shown great promise for the prevention/treatment of T2DM because they are readily available with good safety profiles and various biological activities (8, 9). Recently, more and more studies have shown the alleviation of T2DM by natural polysaccharides via multiple pathways such as regulating insulin resistance, regulation of pancreatic β-cell functions, improvement in glucose metabolism, inhibition of inflammatory pathways, and restoration of intestinal microbiota composition (10, 11).

From a molecular mechanistic perspective, natural polysaccharides significantly attenuate cellular insulin resistance. Astragalus polysaccharides exert therapeutic effects through enhancement of insulin sensitivity, preservation of pancreatic β-cell integrity, and optimization of intestinal microbial communities (12). *Momordica charantia* polysaccharides substantially reduce glycemic levels via activation of the insulin receptor substrate 1 (IRS-1)/phosphatidylinositol 3-kinase (PI3K)/protein kinase B (Akt) and AMP-activated protein kinase (AMPK) signaling pathways (13).

The gut microbiota is one of the important factors responsible for the anti-diabetic activity of polysaccharides. The hypoglycemic effect of Astragalus membranaceus polysaccharide is mediated via the “gut microbiota-mucosal barrier” axis, blocking pathogenic microbes in the gut and favoring growth of probiotics (14). Dendrobium officinale polysaccharides repair intestinal barrier function and modulate gut microbiota structure by regulating the lipopolysaccharide (LPS) /Toll-like receptor 4 (TLR4)/TIR-domain-containing adapter-inducing interferon (TRIF)/nuclear factor-κB (NF-κB) pathway, thus ameliorating the pathology of T2DM (15). Polysaccharides from Cyclocarya paliurus alleviate the symptoms of T2DM via modulating gut microbiota and SCFAs metabolism (16). Taken together, these studies clearly indicate the role of gut microbiota-host metabolism as one of the key mechanisms for polysaccharides to exert antihyperglycemic activity.

The structure–activity correlation between the anti-diabetic bioactivity and structural properties of natural polysaccharides has been observed. The structural parameters, such as monosaccharide composition, molecular mass profile, glycosidic linkage types, and specific functionalities, are all factors that can affect bioactivity (17). However, there is still a lack of systematic studies on the structure–activity relationship for polysaccharides, thus hampering full mechanistic knowledge.

This paper will summarize and critically evaluate some of these latest developments concerning the molecular basis for natural polysaccharides ameliorating T2DM, especially in clarifying the mechanisms of action by which polysaccharides act as hypoglycemic agents via multiple targets related to glucose homeostasis, anti-diabetic effects in the liver, kidney, and muscle; protective effects on pancreatic β-cells; anti-inflammatory activity; antioxidant action; and regulation of gut microflora. In addition to this, in this article we have also discussed the complex structure–activity relationship of polysaccharide structural features with their anti-diabetic activity, which provides a strong evidence base for the clinical application of natural polysaccharides to prevent and treat T2DM.

## 2 Pathophysiological mechanisms of type 2 diabetes mellitus

**Figure 1:**
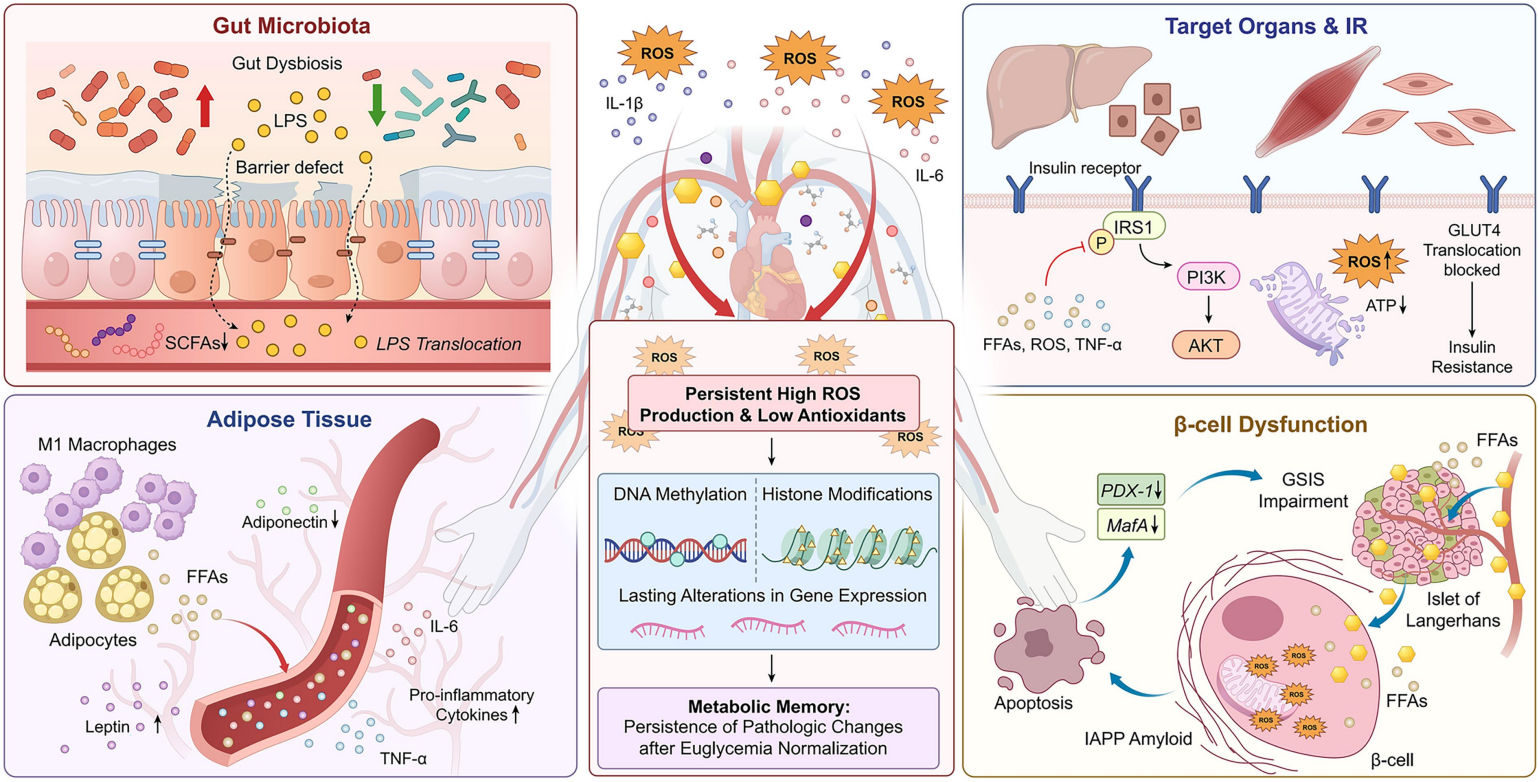
Pathophysiological mechanisms of Type 2 Diabetes Mellitus. The figure illustrates the five principal pathogenic axes of T2DM: insulin resistance, pancreatic β-cell dysfunction, chronic inflammation, oxidative stress, and gut microbiota dysbiosis. These mechanisms are interconnected and mutually reinforcing, collectively disrupting glucose homeostasis and driving disease progression. A dedicated annotation panel for **metabolic memory** is included to highlight the phenomenon whereby prior exposure to hyperglycemia induces persistent epigenetic alterations—including DNA methylation changes and histone modifications—leading to lasting alterations in gene expression that perpetuate oxidative stress and inflammatory signaling even after glycemic control is achieved. This epigenetic “imprinting” underlies the continued elevated risk of diabetic complications following treatment, as discussed in detail in Section 2.4.

Type 2 diabetes mellitus is a complex metabolic disease. Fundamental pathologic processes include IR, pancreatic β-cell dysfunction, chronic inflammation, oxidative stress, and gut microbiota dysbiosis. The interaction of these mechanisms is synergistic, collectively leading to disruption in glucose homeostasis and disease progression. We summarize the pathogenesis of T2DM in detail (Figure 1).

### 2.1 Insulin resistance

The cardinal pathophysiological feature of T2DM is IR, which refers to a diminished biological response of the target tissue to the stimulating effect of insulin (18). The liver, skeletal muscle, and adipose tissue are major target organs of IR, as reduced insulin sensitivity in those tissues leads to impaired glucose uptake and metabolic dysfunction (19).

Insulin signaling transduction pathway defects are also considered to be important mechanisms for the development of IR. Under normal conditions, insulin binds to cell surface insulin receptors, activating insulin receptor tyrosine kinase activity, which then leads to the subsequent activation of insulin receptor substrates (IRS) protein phosphorylation (19, 20). IRS proteins also trigger activation of the PI3K/Akt pathway that eventually leads to GLUT4 translocation to the plasma membrane, thus promoting the entry of glucose into cells (19). Many hubs in that pathway are dysregulated under conditions of IR. Two major forms of metabolic toxicity contribute to the disruption of insulin signaling in peripheral tissues: lipotoxicity and glucotoxicity. Lipotoxicity arises from the chronic elevation of circulating free fatty acids (FFAs) and their ectopic accumulation in non-adipose tissues, particularly skeletal muscle, the liver, and pancreatic β-cells. Excessive FFAs and their intracellular lipid intermediates—including diacylglycerol (DAG) and ceramides—impair insulin signaling by activating specific isoforms of protein kinase C (PKC), which in turn inhibit the phosphorylation and activation of IRS (21, 22). Glucotoxicity, by contrast, stems from sustained hyperglycemia, which progressively deteriorates pancreatic β-cell function and exacerbates insulin resistance; this process is closely linked to oxidative stress and the subsequent activation of the c-Jun N-terminal kinase (JNK) pathway (23). The interplay between lipotoxicity and glucotoxicity establishes a self-perpetuating cycle that progressively aggravates insulin resistance throughout the course of T2DM.

The role of adipose tissue is not only to store energy but also to serve as an important endocrine organ which secretes different adipokine hormones for the regulation of metabolism (24). During obesity and T2DM, the altered production of adipokines is a key factor in the development of IR. Adiponectin is an adipokine with insulin-sensitizing activity that improves insulin sensitivity by activating AMPK (24). Moreover, high concentrations of circulating FFAs may cause IR (25). Beyond general dietary composition, specific functional foods with preserved phytochemical bioactivity have also emerged as promising adjuncts for attenuating insulin resistance. A recent 10-week randomized controlled trial demonstrated that daily supplementation with 20 mL of date (*Phoenix dactylifera*) vinegar significantly improved glycemic and lipid parameters in adults with T2DM and dyslipidemia, lowering HbA1c from 6.85 to 6.08%, LDL-cholesterol from 121.05 to 111.09 mg/dL, and fasting blood glucose from 168.4 to 147.6 mg/dL (*p* < 0.05); complementary molecular docking and dynamics simulations further revealed that its bioactive constituents form stable complexes with key metabolic targets including DPP-IV and SGLT1, supporting a dual-target therapeutic strategy against both glycemic dysregulation and its cardiovascular comorbidities.

Mitochondrial dysfunction has been found to play an important part in the development and progression of IR. The skeletal muscle mitochondrial oxidative phosphorylation capacity is reduced in patients with T2DM (26). Mitochondria are major sources for ROS production, and prolonged oxidative conditions modify mitochondrial functionality, inducing IR and altering glucose tolerance (27). Furthermore, defective mitophagy impairs the removal of abnormal/dysfunctional mitochondria, triggering innate immune/inflammatory pathways that worsen metabolic derangements (28).

### 2.2 Pancreatic β-cell dysfunction

Progressive deterioration of pancreatic β-cell function constitutes a determinative factor in T2DM pathogenesis. Although IR increases insulin demand, overt diabetes develops only when β-cells fail to compensatorily augment insulin secretion (29).

Normal β-cells precisely regulate the level of blood glucose via glucose-stimulated insulin secretion (GSIS), and first-phase GSIS defects are present from the beginning of T2DM, with progressive loss of second-phase secretion and basal insulin secretion in more advanced stages of the disease (30). The impaired function is at least partly due to defective glucose sensing, where reduced glucokinase (GK) activity impacts glucose phosphorylation, which is the rate-limiting step (31).

Recent investigations have revealed that β-cell functional loss does not solely result from cell death; cellular phenotype conversion represents an important mechanism (4). Under hyperglycemic and hyperlipidemic conditions, expression of mature β-cell-specific transcription factors such as pancreatic and duodenal homeobox 1 (PDX-1) and musculoaponeurotic fibrosarcoma oncogene homolog A (MafA) declines, causing β-cells to lose their mature phenotype and express progenitor cell markers, thereby undergoing dedifferentiation (4). Some dedifferentiated β-cells can transdifferentiate into *α*-cells (32), resulting in reduced β-cell numbers and increased glucagon secretion. This cellular fate conversion may be reversible, providing theoretical foundations for diabetes remission (33).

Loss of β-cell mass/function is an important part of T2DM pathology. By the time of T2DM diagnosis, up to half (i.e., ~40–50%) of β-cell function may be lost, with a further loss of 4–5% per year subsequently (29). There are many causes of β-cell damage such as glucotoxicity, lipotoxicity, inflammation, oxidative stress, and ER stress (34). IAPP accumulation is a pathological feature in T2DM. Cytotoxic fibrillary IAPP deposits trigger apoptosis-mediated loss of pancreatic β-cells, leading to the onset of β-cell failure and T2DM (35). Also, pro-inflammatory cytokines, including interleukin-1β (IL-1β) and tumor necrosis factor-*α* (TNF-α), can also drive β-cell apoptosis (36).

Cluster of differentiation 36 (CD36) is an important fatty acid transporter protein that contributes to β-cell dysfunction; hyperglycemia stimulates its upregulation, enhancing FA uptake, amplifying oxidative stress (OS), and inducing β-cell apoptosis. CD36-mediated OS impairs crucial transcription factors (PDX-1 and MafA), which eventually leads to the dysfunction of beta-cells (37). The glutathione (GSH) antioxidant pathway plays a critical role in preserving β-cell viability. GSH is decreased during the course of T2DM, leading to reduced antioxidant capacity with increased vulnerability of β-cells to oxidative damage (38). Therefore, inhibition of CD36 along with improved antioxidant capacity could be a potential approach toward protecting β-cells from damage.

### 2.3 Chronic inflammatory response

Chronic low-grade inflammation represents a prominent feature of T2DM, permeating the entire disease development process. This inflammatory state not only participates in IR and β-cell dysfunction but also promotes diabetic complication occurrence (39).

Sera from patients suffering from T2DM present high concentrations of pro-inflammatory cytokines. The pro-inflammatory cytokine TNF-*α* is related to IR and metabolism, maintaining the immune microenvironment in an inflammatory status and exacerbating metabolic inflammation in T2DM (40). TNF-α can also damage β-cells, leading to islet cell dysfunction (41). IL-6 represents another main pro-inflammatory factor, and its increase is strongly related to a higher incidence rate for T2DM. The combined use of IL-6 and CRP could be used to predict T2DM risk and suggests that there is a synergistic effect between inflammatory factors (42). IL-6 promotes hepatic gluconeogenesis and IR by activating signal transducer and activator of transcription 3 (STAT3) signaling (43, 44). IL-1β is especially relevant to β-cell failure. β-cells can also synthesize IL-1β by themselves and create a self-sustaining autocrine cycle of inflammation and nitric oxide (NO) generation, which decreases mitochondrial ATP levels and eventually leads to beta-cell failure and reduced insulin secretion (45).

T2DM is accompanied by immune cell functional and proportional dysregulation. Adipose tissue macrophage metabolic dysregulation represents a pivotal event in obesity and T2DM (46). Macrophages transition from anti-inflammatory M2 type to pro-inflammatory M1 type, secreting abundant inflammatory factors including TNF-*α* and IL-6, intensifying local and systemic inflammation, and directly or indirectly inhibiting insulin signal transduction, thereby playing critical roles in insulin resistance (47, 48). The adaptive immune system also participates in T2DM pathogenesis. Increased T helper 17 (Th17) cells concurrent with decreased regulatory T cells (Tregs) lead to pro-inflammatory/anti-inflammatory imbalance (49). Different innate lymphocyte, T cell, and B cell subpopulations exert varying effects on glucose homeostasis, suggesting that alterations in immune cell distribution may play important roles in T2DM development (50). Furthermore, T2DM patients exhibit hypercoagulable states and coagulation abnormalities, which are also closely related to chronic inflammation (51).

### 2.4 Oxidative stress

Oxidative stress is defined as an imbalance between the production of reactive oxygen species (ROS) and antioxidant defenses that plays a pivotal role in the pathophysiology of T2DM and its complications (52). The main mechanisms involved in hyperglycemia-induced increase in ROS are glucose auto-oxidation and mitochondrial respiratory chain overactivity induced by hyperglycemia with consequent O₂^−^ production. Leakage of electrons from the electron transport chain complexes I and III combine with oxygen molecules to produce O₂^−^ that in turn converts into hydrogen peroxide (H₂O₂) and hydroxyl radicals (·OH) (53).

The use of a large amount of reduced nicotinamide adenine dinucleotide phosphate (NADPH) for the polyol pathway results in inhibition of GSH generation, reducing antioxidant capacity (54). Low NADPH/NADP^+^ ratio decreases cellular antioxidant capacity, which leads to the production of ROS (55). Hyperglycemia can also lead to the generation of advanced glycation end products (AGEs). Binding between AGE and its receptor (RAGE) induces activation of NADPH oxidase, greatly augmenting ROS generation (56). Furthermore, the hexosamine pathway is linked with insulin resistance and hyperglycemia development. The hexosamine pathway could induce transforming growth factor β (TGF-β) encoding, increasing ROS generation, repressing the expression of antioxidant enzymes, and facilitating redox imbalance. On the other hand, ROS upregulates TGF-β expression, creating a vicious circle of oxidative stress with hyperglycemia (57).

The activity of antioxidant enzymes such as SOD, CAT, and GPx decreases in T2DM patients (58, 59). Nuclear factor erythroid 2-related factor 2 (Nrf2) is a transcription factor that regulates antioxidant genes. Oxidative stress-induced activation of the Nrf2 pathway leads to increased expression of antioxidants (60). However, chronic stimulation of the Nrf2 pathway causes metabolic changes that induce a reductive stress state, leading to disease development (61). Targeting the Nrf2 axis has therefore attracted growing interest as a nutraceutical strategy against oxidative stress–driven metabolic and neurological complications. In line with this, a recent peptidomic and molecular-docking study identified three novel oat protein–derived peptides — DFVADHPFLF (DF-10), HGQNFPIL (HL-8), and RDFPITWPW (RW-9) — that enhanced antioxidant enzyme activity and suppressed ROS accumulation by upregulating the Nrf2-Keap1/HO-1 pathway both *in vitro* and *in vivo*; in a zebrafish model, these peptides further attenuated MDA levels, AChE activity, and pro-inflammatory cytokines while elevating *Bdnf*, *Nrf2*, and *Egr1* mRNA expression, highlighting the translational potential of food-derived bioactive peptides in counteracting the oxidative and inflammatory sequelae of chronic hyperglycemic states.

Oxidative stress also contributes to insulin resistance. ROS activates stress-sensitive serine kinases, including JNK, blocking the action of IRS-1 and inhibiting the transduction of the insulin signal (62). ROS damage also affects mitochondria, diminishing ATP production and impairing GSIS efficiency (63). Apoptosis of β-cells occurs in response to oxidative stress via the mitochondria, releasing cytochrome c and triggering the caspase cascade of apoptosis (64).

The “metabolic memory” phenomenon describes the persistence of hyperglycemia-induced pathologic changes after normalizing blood glucose levels. Oxidative stress is a relevant pathway involved in this process (65). A transient exposure to high glucose may lead to sustained elevation of ROS production and impaired antioxidant systems, continually changing the pattern of gene expression via epigenetic processes, including DNA methylation and modification of histones (66) (Figure 1; Oxidative Stress & Metabolic Memory Module). This metabolic memory highlights the need for early aggressive glycemic control and implies that glycemic improvement alone might not be sufficient to fully reverse oxidative injury, requiring the combination of antioxidant treatments.

### 2.5 Gut microbiota dysbiosis

Gut microbiota is a collective term for the microbes inhabiting the gastrointestinal tract, and they are responsible for performing several functions related to metabolism. Dysbiosis of gut microbiota has been found to be directly associated with the development of T2DM (67). There is a marked reduction in alpha-diversity along with specific compositional changes of gut microbiota in patients suffering from T2DM. There occurs an imbalance between Firmicutes and Bacteroidetes, butyrate-producing bacteria decline, and opportunistic pathogens rise (68).

The gut microbiota regulates host metabolism by producing metabolites, such as SCFAs, which are derived from fermenting dietary fiber, mainly acetate, propionate, and butyrate (69). SCFAs enhance metabolism via several pathways, such as G protein-coupled receptor activation, intestinal barrier function improvement, inhibiting inflammation, and inducing the development of regulatory T cells (70). Bacteria that produce SCFAs decrease in T2DM patients (71), and lower SCFAs levels weaken the above-mentioned protection. Dietary interventions rich in fermentable fiber can reverse this SCFAs deficit and restore downstream protection. For instance, oat bran supplementation was shown to selectively enrich SCFAs-producing probiotic genera and elevate circulating SCFAs in high-fat-diet–fed mice, concomitantly suppressing systemic pro-inflammatory cytokines and lowering inflammation-associated circulating metabolites — effects mechanistically linked to MAPK signaling modulation. Metabolic anomalies of bile acids (BA) are also involved in T2DM, as gut microbiota regulate BA composition via bile salt hydrolase, affecting farnesoid X receptor (FXR) activation (72). FXR has regulatory roles in glucose-lipid metabolism, bile acid synthesis and secretion, inflammation, intestinal permeability, and the regulation of insulin sensitivity (73); microbiota dysbiosis causes BA metabolism disorder and FXR signaling dysfunction (74). Trimethylamine N-oxide (TMAO) is a metabolite derived from the gut microbiota. TMAO is associated with a higher risk of IR and T2DM, which may be mediated by some pathways like endoplasmic reticulum stress (75).

The intestinal epithelial barrier, formed by a monolayer of epithelial cells with tight junction proteins to prevent leakage of intestinal lumen contents (76), is disrupted in microbiota dysbiosis through disruption of tight junction protein expression, increasing intestinal permeability (77, 78). A damaged intestinal barrier permits the passage of LPS from gram-negative bacterial cell walls to circulate, inducing the condition known as metabolic endotoxemia. Low levels of LPS can also activate the NF-κB pathway via TLR4 and cause chronic low-grade inflammation, which in turn affects insulin signal transduction, which promotes IR and β-cell dysfunction (78). Beyond microbial composition, fiber-mediated remodeling of host metabolism also contributes to alleviating this inflammation–insulin resistance axis. A follow-up investigation further demonstrated that oat bran intake, particularly when combined with moderate-intensity exercise, intensified carbohydrate and lipid metabolism — reflected in altered levels of lactate, pyruvate, succinate, fumarate, malate, and citrate — while simultaneously boosting antioxidant capacity (SOD, GSH) and curbing inflammation-related circulating metabolites, underscoring the integrative role of dietary fiber in restoring gut–metabolic–immune homeostasis.

The gut microbiota modulates glucose metabolism via the gut–brain–pancreas axis. Microbiota metabolites control intestinal endocrine cells’ secretion of incretins, including GLP-1, enhancing insulin secretion and improving insulin sensitivity (79). Microbiota also send signals to the brain through the vagus nerve, which influence hunger (80), as well as have regulatory effects on energy metabolism (81) and glucose regulation (82). The dysbiosis of the microbiota disturbs all this regulation and leads to metabolic disorder. In addition, the microbiota regulates epigenetic modifications (83), playing a role in the progression of T2DM (84). Multi-omics evidence further extends this gut-brain dialogue to dietary bioactives: in scopolamine-challenged mice, the oat protein–derived peptide RW-9 restored the abundance of *Muribaculaceae*, *Lachnospiraceae*, and *Lactobacillus*, elevated circulating neurotransmitters (5-HT, dopamine, arginine) and ABC-transporter expression, and activated hippocampal PI3K/Akt, cAMP and JAK–STAT signalling — a constellation of microbiota–metabolite–host interactions that illustrates how food-derived bioactives can rewire the gut–brain–metabolism axis that is increasingly recognized as a determinant of the neurovascular and cognitive sequelae of chronic hyperglycaemia.

## 3 Sources and classification of natural polysaccharides

Natural polysaccharides are natural biomacromolecules consisting of at least 10 monosaccharide residues connected via glycosidic bonds, showing a wide distribution among plants, fungi, animals, and marine organisms. According to the sources of origin, natural polysaccharides could be classified into four main groups: plant-derived polysaccharides, fungal-derived polysaccharides, animal-derived polysaccharides, and marine-derived polysaccharides.

### 3.1 Plant-derived polysaccharides

Polysaccharides from plants mainly come from the root, stem, leaf, fruit, and seed parts of medicinal plants, which are a kind of heteropolysaccharide consisting of many kinds of monosaccharides with immune regulation and other pharmacological activities (antitumor action, antioxidant effect, hypoglycemic activities). Some representatives of the plant polysaccharides are:

*Astragalus membranaceus* is one of the most important qi-tonifying herbs in TCM and has astragaloside IV as its major active component. Astragalus polysaccharides (APS) are a group of astragalus water-soluble heteropolysaccharides (85). Most APS are heteropolysaccharides, including dextrose, neutral polysaccharides, and acidic polysaccharides, where the alpha type of glycosidic linkage is dominant (86). The structure–activity relationship of APS shows that there is a close connection between them. Certain MW, monosaccharide composition ratio, and glycosidic linkage pattern are important structural factors determining the immunological properties of APS (85). The mechanism by which APS modulates immune function is via specific interaction with TLR4, activating downstream signaling cascades (87). APS alleviates diabetes by improving insulin resistance, regulating the immune system, protecting pancreatic islet cells, and promoting the improvement of gut microbial communities (12). Additionally, APS has also been shown to be a neuroprotectant that ameliorates neurodegenerative disease via antioxidant and anti-inflammatory effects (88) and, in addition, modulates gut microbiota for maintaining intestinal health (89).

The fruit of *Lycium barbarum* is rich in *Lycium barbarum* polysaccharide (LBP), which consists mainly of arabinose, glucose, and galactose (90). LBP has a strong antioxidant capacity, scavenges free radicals, and increases the activity of antioxidant enzymes such as SOD, CAT, and GPx (91). In diabetes treatment, LBP decreases the level of blood sugar and improves insulin resistance (92). LBP provides retinal protection, preventing diabetic retinopathy (93).

Polysaccharides of ginseng have immunomodulatory properties (94). Polysaccharides from *Portulaca oleracea* exhibit activities to attenuate inflammation and oxidative stress, inhibiting pathogens, enhancing intestinal mucosal barriers, and maintaining gut homeostasis (95). Beyond these well-characterized species, structurally novel pectin-like polysaccharides have also been identified from traditional medicinal plants. For example, two high-molecular-weight pectin-like fractions recently isolated from *Gastrodiae rhizoma* — GRP40-F1 and GRP75-F1, composed primarily of rhamnose, galactose, glucose, mannose, and galacturonic acid — significantly suppressed nitric oxide and prostaglandin E2 production in LPS-stimulated RAW 264.7 macrophages (IC50 of 90.89 and 86.80 μg/mL, respectively), with GRP75-F1 dose-dependently down-regulating phosphorylated p38, ERK, and JNK without altering total protein levels, thereby implicating MAPK pathway inhibition as a central anti-inflammatory mechanism. These plant polysaccharides are composed of complex structures, have extensive biological activities, and minimal toxic side effects, providing abundant resources for novel drug development.

### 3.2 Fungal-derived polysaccharides

Polysaccharide is one of the main active components of edible and medicinal fungi; β-glucan is its typical structural component, which has unique immunological effects and anti-cancer activity (96).

*Ganoderma lucidum* is an ancient medicinal fungus, and the main active ingredient in it—*Ganoderma lucidum* polysaccharides (GLP)—is among the most important bioactive compounds. The main backbone of GLP is β-D-glucan connected through β-(1 → 3) and β-(1 → 6) glycosidic linkages, with which such a special architecture constitutes the structural basis of its activity (97). GLP stimulates cell and humoral immunity through activation of dendritic cells, macrophages, and natural killer cells (98). GLP also has pharmacologic effects such as hypoglycemic, hypolipidemic, antiviral, and antiallergic effects (99), as well as ameliorative effects on T2DM and insulin resistance states (100).

The biological activity of lentinan includes anti-inflammatory, antiviral, immunomodulatory, and antioxidant effects, with proven ability in protecting pancreatic β-cells (101). The *Grifola frondosa* polysaccharides have anti-obesity effects, attenuate chronic inflammation of adipose tissue and liver by inhibiting the TLR4/NF-κB signaling pathway (102). Polysaccharides from *Inonotus obliquus* possess strong antioxidant, antibacterial, and immune-modulating activity (103), while polysaccharides of *Cordyceps sinensis* are excellent for antioxidant as well as anti-diabetic aspects (104).

### 3.3 Animal-derived polysaccharides

Animal polysaccharides mainly come from the exoskeletons of arthropods, cartilage tissue, or connective tissue, with chitosan and chondroitin sulfate being the representative ones, which have unique biocompatibility and biodegradability, finding wide applications in biomedical materials, drug delivery systems, etc. (105).

Chitosan is mainly obtained from the exoskeletons of crustaceans, including shrimps and crabs. Chitosan consists of polysaccharides made up of D-glucosamine and N-acetyl-D-glucosamine connected with a β-(1 → 4)-glycosidic bond (106). Degree of substitution and molecular weight are important parameters that influence the characteristics and biological activity of chitosan (107). Chitosan has good biocompatibility, biodegradability, nontoxicity, and antibacterial activity and is widely used in drug delivery applications (108). Chitosan hydrogels have shown potential for promoting angiogenesis, promoting cell proliferation, and suppressing inflammation, which is widely used for treating diabetic wounds (109).

Chondroitin sulfate (CS) is a natural viscous polysaccharide that mainly exists in tissues like cartilage, and it is one of the important components in the cartilage extracellular matrix, having anti-inflammatory, hypolipidemic, and immunomodulatory properties (110). Lithium chondroitin sulfate hydrogels can ameliorate osteogenic defects in the diabetic microenvironment via modulation of macrophage polarization, related to the activation of the glycogen synthase kinase-3β/β-catenin pathway (111). In addition, chondroitin sulfate has a preventative effect on STZ-diabetic osteoporosis by reducing the level of glucose, anti-oxidative stress, anti-inflammatory effects, and OPG/RANKL expressions (112).

Hyaluronic acid (HA), which is distributed in connective tissue and vitreous body, has functions such as retaining water, lubrication, anti-inflammation, and antioxidant activities (113). The biological activity of HA is intimately correlated to its MW; high MW HA has anti-inflammatory, immunosuppressive, as well as tissue-protecting actions, whereas low molecular weight HA displays pro-inflammatory activities (114). Studies showed that hyaluronic acid self-healing hydrogel has good prospects for diabetic wound healing (115). Systematic review and meta-analysis found that hyaluronic acid and its derivatives can increase the healing rate of DFU and shorten the healing time (116).

### 3.4 Marine-derived polysaccharides

Marine biodiversity has rich resource development prospects in marine polysaccharides. Marine polysaccharides mainly come from algae and sea cucumber, which have different chemical structures as well as different bioactivities (117).

Polysaccharides are novel compounds isolated from algae, which have the effect of protecting against diabetes mellitus and antioxidant injuries, which are involved, at least partly, in the protection of RPE cells against HG injury *in vitro* (118). Sodium alginate (SA) is a natural polysaccharide extracted from brown algae, which can ameliorate the development of DN via regulating glucose-lipid metabolism and blocking inflammation, upregulating miR-146a levels, controlling the oxidative response, and inhibiting apoptosis (119). Marine fucosylated polysaccharides can regulate blood glucose levels by inhibiting the activity of digestive enzymes, improving insulin sensitivity, beta-cell preservation, and intestinal flora regulation (120).

Sea cucumber represents a traditional nourishing food and medicinal material, with sea cucumber polysaccharides constituting one of its important active constituents. Sea cucumber polysaccharides can regulate gut microbiota composition, increase SCFAs-producing bacteria generation, reduce opportunistic pathogen production, improve glucose tolerance, and alleviate T2DM symptoms (121). *Ruditapes philippinarum* polysaccharides can also modulate gut microbiota composition, increase beneficial bacteria presence, ameliorate glucose metabolism indicators, and improve hyperglycemic symptoms (122).

### 3.5 Extraction and purification methods

**Figure 2:**
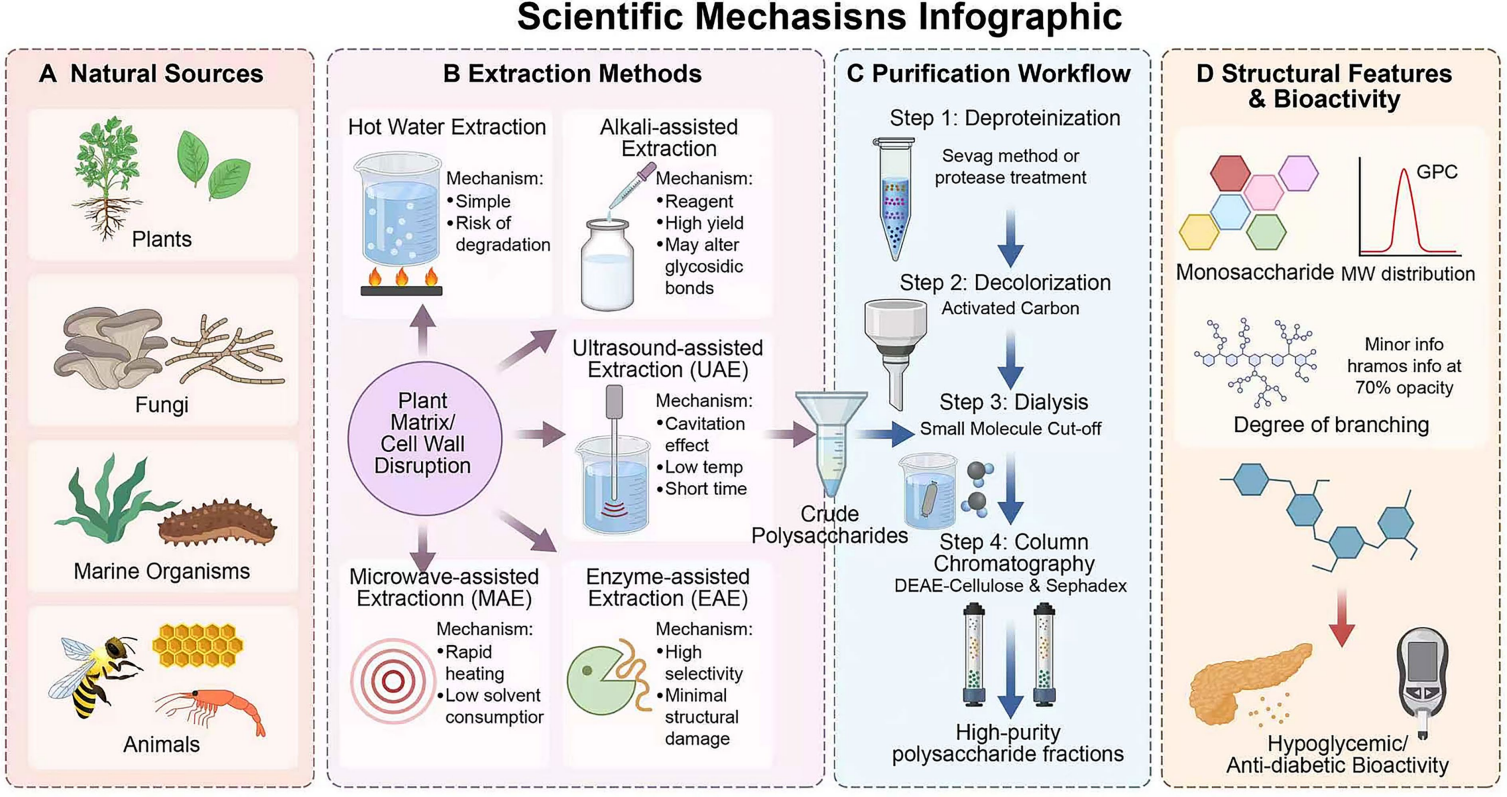
Major natural sources of anti-diabetic polysaccharides and representative extraction workflows. The schematic illustrates four primary categories of polysaccharide sources—plants (roots, leaves, seeds, and fruits), fungi (mushrooms and mycelium), marine organisms (seaweed, sea cucumbers), and animals (bee products, shellfish)—alongside a generalized extraction and purification workflow. Key extraction technologies depicted include hot water extraction, alkali-assisted extraction, ultrasound-assisted extraction (UAE), enzyme-assisted extraction (EAE), and microwave-assisted extraction (MAE). The figure highlights how extraction conditions and purification steps influence final polysaccharide structural features, which in turn determine anti-diabetic bioactivity.

As shown in Figure 2, the structural integrity and biological activity of polysaccharides are profoundly influenced by the extraction and purification methods employed. Hot water extraction remains the most widely used approach for its operational simplicity and broad applicability; however, prolonged high-temperature exposure may cause depolymerization and structural degradation, reducing molecular weight and potentially diminishing bioactivity. Alkali-assisted extraction enhances yields for tightly bound polysaccharides but may alter glycosidic linkages and introduce structural artifacts. Ultrasound-assisted extraction (UAE) employs acoustic cavitation to disrupt cell walls at lower temperatures, yielding polysaccharides with better-preserved molecular weight profiles and shorter extraction times. Enzyme-assisted extraction (EAE) utilizes cellulases, pectinases, or proteases to facilitate polysaccharide release from the plant matrix with high selectivity and minimal structural degradation. Microwave-assisted extraction (MAE) enables rapid heating of the extraction medium, improving efficiency and reducing solvent consumption. Following crude extraction, sequential purification steps—including deproteinization (Sevag method or protease treatment), decolorization (activated carbon), dialysis, and column chromatography (DEAE-cellulose anion exchange and Sephadex gel filtration)—are typically employed to obtain high-purity polysaccharide fractions suitable for structural and biological characterization. The choice of extraction and purification protocol should be guided by the target source material and the intended downstream application, as these parameters directly modulate monosaccharide composition, molecular weight distribution, and degree of branching—all of which are critical determinants of hypoglycemic bioactivity.

## 4 Molecular mechanisms by which natural polysaccharides ameliorate type 2 diabetes mellitus

**Figure 3:**
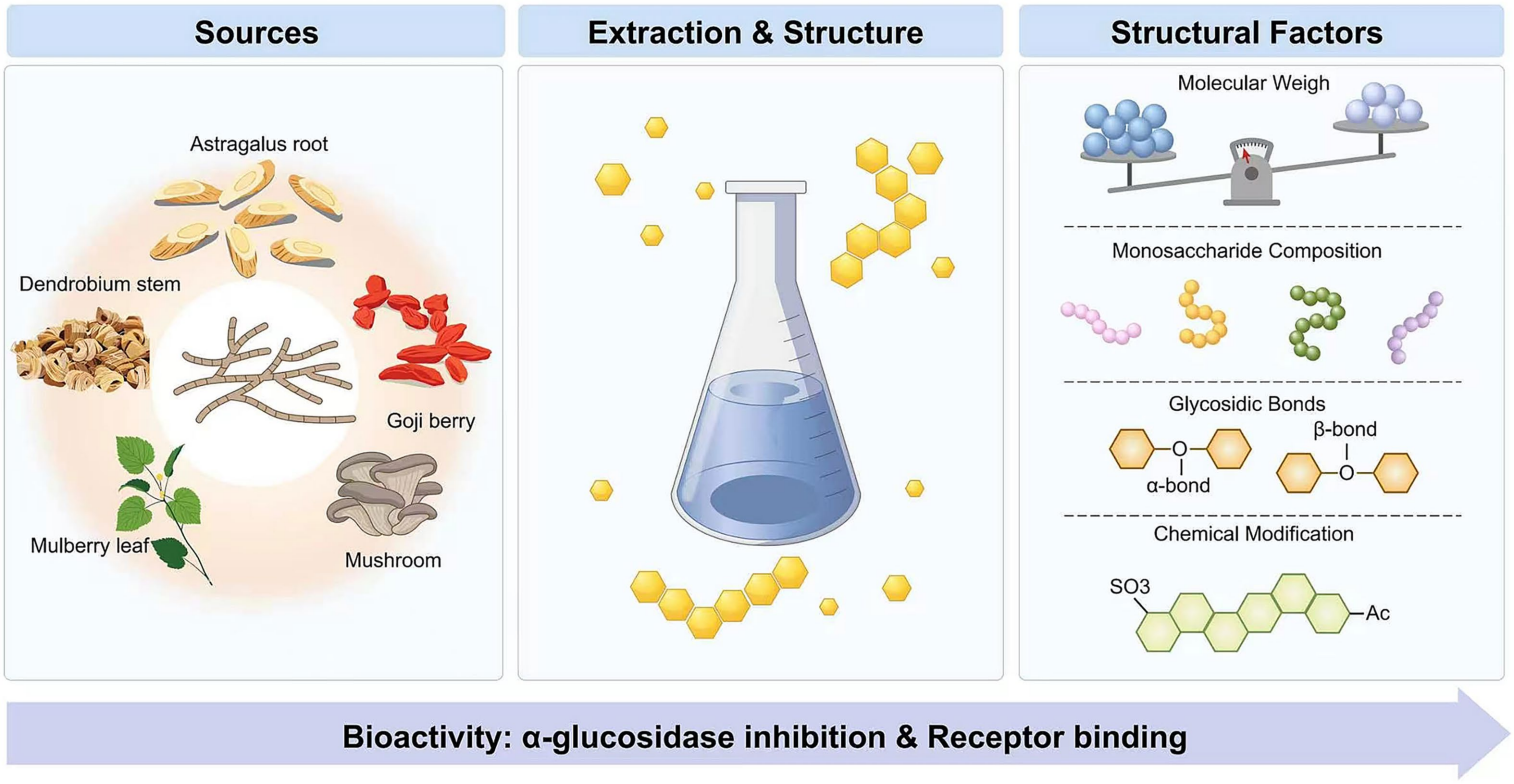
Sources and structural determinants that affect anti-diabetic activity of natural polysaccharides. Natural polysaccharides extracted from medicinal plant (e.g., *Astragalus membranaceus*, *Lycium barbarum*) or fungus have hypoglycemic effect. Their biological activity depends exclusively on their structural properties (molecular weight, monosaccharide composition, glycosidic linkages (α/β)) and chemical modifications that together dictate how they block digestion and attach themselves to receptors on the cell surface.

T2DM is a multifactorial disease, and its etiopathogenesis includes, among others, the development of insulin resistance, pancreatic β-cell failure, chronic inflammation, oxidative stress, and dysbiosis of the gut microbiota (12, 123). Natural polysaccharides, as a class of extensively accessible and highly safe bioactive macromolecules, exert multi-target and multi-pathway regulating effects on the alleviation of T2DM (124) (see Table 1; Figure 3). In this chapter, we will describe in detail how these naturally occurring polysaccharides alleviate T2DM by regulating glucose metabolism, pancreatic β-cell protective effects, anti-inflammatory properties, reduction of oxidative stress, and modulation of the intestinal microbiota.

**Table 1:** Molecular mechanisms by which natural polysaccharides ameliorate type 2 diabetes mellitus.

Agents	Source	Model	Key molecular changes	Pathway affected	Target mechanism	References
*Astragalus mongholicus* polysaccharides	*Astragalus mongholicus*	HSHFD and STZ-induced SD rats	PI3K↑, Akt↑, GLUT4↑	PI3K/Akt	Enhancement of Insulin Signaling Pathways	(125)
*Momordica charantia* l. polysaccharides	*Momordica charantia* l.	HFD and STZ-induced C57BL/6 mice	p-IRS1/IRS1↓, p-PI3K/PI3K↑, p-Akt/Akt↑, GLUT4↑, p-GSK-3β↑, FoxO1↓, p-AMPK/AMPK↑, PPARα↑, PGC1α↑, SIRT1↑	IRS1/PI3K/Akt, AMPK	Enhancement of Insulin Signaling Pathways	(13)
Coix seed polysaccharides	Coix seed	HFD and STZ-induced C57BL/6 J mice	IGF1↑, IGF1R↑, IRS↑, PI3K↑, Akt↑	IGF1/PI3K/Akt	Enhancement of Insulin Signaling Pathways	(126)
Selenium-enriched cordyceps militaris polysaccharides	Cordyceps militaris	HepG2 cells	p-Akt/Akt↑, GLUT4↑, p-PI3K/PI3K↑	PI3K/Akt	Promotion of Peripheral Tissue Glucose Transport	(127)
Gynura divaricata polysaccharides	Gynura divaricata	HSHFD and STZ-induced C57BL/6 mice	p-PI3K↑, p-Akt↑, GLUT-4↑	PI3K/Akt/GLUT-4	Promotion of Peripheral Tissue Glucose Transport	(128)
Dendrobium officinale polysaccharides	Dendrobium officinale	HFD and STZ-induced C57BL/6 J mice	p-Akt/Akt↑, p-FoxO1/FoxO1↑, G6Pase↓, PEPCK↓, PKA-C↓, p-PKA↓	cAMP-PKA, Akt/FoxO1	Modulation of Hepatic Glucose Metabolism	(130)
*Astragalus mongholicus* polysaccharides	*Astragalus mongholicus*	Transport -induced one-day-old chicks	PGC-1α↑, SIRT1↓, p-AMPK/AMPK↑, PPARγ↓	PGC-1α/SIRT1/AMPK/PPARα/PPARγ	Modulation of Hepatic Glucose Metabolism	(131)
Rehmannia glutinosa polysaccharides	Rehmannia glutinosa	STC-1 cells	PI3K↑, p-Akt↑	PI3K/Akt	Inhibition of Digestive Enzyme Activities	(135)
Opuntia milpa alta polysaccharides	Opuntia milpa alta	INS-1 cells	Bcl-2↑, Bax↓, caspase-3↓, caspase-9↓, cleaved caspase-3↓, cleaved PARP↓	Nrf2	Anti-Apoptotic Effects	(138)
Lentinus edodes mycelia polysaccharides	Lentinus edodes mycelia	INS-1 cells	Bax/Bcl-2↓, cleaved caspase-3↓, cleaved caspase-1↓, p-p38/p38↓, p-JNK/JNK↓, Nucleus p65↓, Nucleus Nrf2↑	p38 MAPK, JNK, NF-κB, Nrf2	Anti-Apoptotic Effects	(139)
Bee pollen polysaccharide from *rosa rugosa* thunb. (Rosaceae)	*Rosa rugosa* thunb. (Rosaceae)	MIN6 cells, alloxan-induced C57BL/6 J mice	p-p38/p38↑, p-Erk/Erk↑, p-Akt/Akt↑	Akt, MAPK	Enhancement of Insulin Secretion	(141)
Squash polysaccharides	Squash	MIN6 cells	ATP↑, Ca^2+^↑, Caspase-3↓	—	Enhancement of Insulin Secretion	(142)
*Momordica charantia* polysaccharide	*Momordica charantia*	STZ-induced SD rats	NF-κB↓, Caspase-3↓	NF-κB	Suppression of NF-κB Signaling Pathway	(145)
*Lycium barbarum* polysaccharide	*Lycium barbarum*	HFD and STZ-induced SD rats	MCP-1↓, IL-18↓, NF-κB↓, NLRP3↓, FGFR4↓, FXR↑, FGF15↓	FXR-FGF15-FGFR4, NF-κB	Suppression of NF-κB Signaling Pathway	(146)
Gynostemma pentaphyllum herb. Polysaccharides	Gynostemma pentaphyllum herb.	STZ-induced Kunming mice	IL-4↑, IL-10↑, TNF-α↓, IL-6↓	—	Modulation of Inflammatory Factor Balance	(148)
Pseudostellaria heterophylla polysaccharides	Pseudostellaria heterophylla	HFD and STZ-inducedWistar rats	IL-10↑, TNF-α↓	—	Modulation of Inflammatory Factor Balance	(149)
Panax notoginseng leaves polysaccharides	Panax notoginseng leaves	HepG2 cells	CAT↑, SOD↑, GSH-Px↑, MDA↓	—	Enhancement of Antioxidant Enzyme Activities	(151)
*Astragalus mongholicus* polysaccharides	*Astragalus mongholicus*	HSHFD and STZ-induced SD rats	GSH↑, GSH-Px↑, SOD↑, MDA↓	—	Enhancement of Antioxidant Enzyme Activities	(152)
Lentinus edodes polysaccharides	Lentinus edodes	HSHFD and STZ-induced Kunming mice	MDA↓, SOD↑, GSH↑, Nrf2↑, HO-1↑	Nrf2/HO-1	Activation of Nrf2 Pathway	(155)
Ganoderma applanatum polysaccharides	Ganoderma applanatum	HSHFD and STZ-induced Kunming strain mice	Bax↓, Caspase-3↓, Bcl-2↑, TLR4↓, MyD88↓, p-NF-κB P65/NF-κB P65↓, SOD↑, CAT↑, GSH-Px↑, Nrf2↑, HO-1↑, Keap1↓	Nrf2/Keap1-TLR4/NF-κB-Bax/Bcl-2	Activation of Nrf2 Pathway	(156)
*Astragalus mongholicus* polysaccharides	*Astragalus mongholicus*	HSHFD and STZ-induced SD rats	Restore Firmicutes/Bacteroidetes (F/B) ratio, Clostridia↓, Proteobacteria↓, PI3K↑, Akt↑, GLUT4↑, TLR4↓, p-IκB/IκB↓, p-NF-κB/NF-κB↓	PI3K/Akt, TLR4/NF-kB	Improvement of Microbiota Structure	(125)
*Astragalus mongholicus* polysaccharides	*Astragalus mongholicus*	HFD and STZ-induced C57BL/6 J mice	Shigella↓, *Allobaculum*↑, Lactobacillus↑	—	Improvement of Microbiota Structure	(14)
Dendrobium officinale polysaccharide	Dendrobium officinale	HFD and STZ-induced C57BL/6 J mice	*Helicobacter pylori*↓, *Allobaculum*↑, Bifidobacterium↑, Lactobacillus↑, TLR4↓, TRAM↓, TRIF↓, p-IKKβ↓, p-p65↓	LPS/TLR4/TRIF/NF-kB	Improvement of Microbiota Structure	(15)
Cyclocarya paliurus polysaccharides	Cyclocarya paliurus	HFD and STZ-induced SD rats	SCFAs-producing bacteria↑, SCFAs↑, GPR41↑, GPR43↑, GPR109a↑	—	Improvement of Microbiota Structure	(16)
Polygonati rhizoma polysaccharide	Polygonati rhizoma	—	SCFAs↑, GPCR41↑, GPCR43↑	PI3K/Akt, TLR4/MyD88/NF-κB	Promotion of SCFAs Generation	(163)
Ruditapes philippinarum polysaccharides	Ruditapes philippinarum	HSHFD and STZ-induced ICR mice	SCFAs↑	—	Promotion of SCFAs Generation	(122)
Fructus mori polysaccharides	Fructus mori	HFD and STZ-induced C57BL/6 J mice	Claudin-1↑, Occludin↑, ZO-1↑, TLR4↓, MyD88↓, p-IKKβ↓, p-p65↓	TLR4/MyD88/NF-κB	Restoration of Intestinal Barrier Function	(166)
Polygonatum kingianum polysaccharides	Polygonatum kingianum	HFD-induced SD rats	Occludin↑, ZO-1↑, TLR4↓, IκB-α↑	TLR4/NF-κB	Restoration of Intestinal Barrier Function	(167)

This table summarizes representative natural polysaccharides reported to alleviate T2DM, together with their botanical/biological sources, experimental models, key molecular changes, signaling pathways involved, and the proposed target mechanisms.

Arrows denote the direction of change relative to the diabetic/model control: ↑, up-regulated or increased; ↓, down-regulated or decreased. The prefix “p-” indicates the phosphorylated form of a protein; “p-X/X” denotes the ratio of the phosphorylated form to the total protein.

Animal/cell models: HFD, high-fat diet; HSHFD, high-sugar high-fat diet; STZ, streptozotocin; SD, Sprague–Dawley; ICR, Institute of Cancer Research; HepG2, human hepatoma cell line; INS-1 and MIN6, rat/mouse pancreatic β-cell lines; STC-1, murine enteroendocrine cell line.

Molecules and pathways: PI3K, phosphoinositide 3-kinase; Akt, protein kinase B; IRS1, insulin receptor substrate 1; IGF1/IGF1R, insulin-like growth factor 1/receptor; GLUT4, glucose transporter 4; GSK-3β, glycogen synthase kinase 3β; FoxO1, forkhead box O1; AMPK, AMP-activated protein kinase; PPARα/γ, peroxisome proliferator-activated receptor α/γ; PGC-1α, PPARγ coactivator 1α; SIRT1, sirtuin 1; G6Pase, glucose-6-phosphatase; PEPCK, phosphoenolpyruvate carboxykinase; cAMP, cyclic adenosine monophosphate; PKA, protein kinase A; Bcl-2/Bax, B-cell lymphoma 2/Bcl-2-associated X; PARP, poly(ADP-ribose) polymerase; MAPK, mitogen-activated protein kinase; JNK, c-Jun N-terminal kinase; Erk1/2, extracellular signal-regulated kinase 1/2; NF-κB, nuclear factor κB; IκB/IKKβ, inhibitor of κB/IκB kinase β; TLR4, Toll-like receptor 4; MyD88, myeloid differentiation primary response 88; TRAM/TRIF, TRIF-related adaptor molecule/TIR-domain-containing adapter-inducing interferon-β; NLRP3, NLR family pyrin domain containing 3; FXR, farnesoid X receptor; FGF15/FGFR4, fibroblast growth factor 15/receptor 4; MCP-1, monocyte chemoattractant protein 1; IL-4/6/10/18, interleukin-4/6/10/18; TNF-α, tumor necrosis factor α; CAT, catalase; SOD, superoxide dismutase; GSH, glutathione; GSH-Px, glutathione peroxidase; MDA, malondialdehyde; Nrf2, nuclear factor erythroid 2-related factor 2; HO-1, heme oxygenase 1; Keap1, Kelch-like ECH-associated protein 1; SCFAs, short-chain fatty acids; GPR41/43/109a, G-protein-coupled receptor 41/43/109a; ZO-1, zonula occludens-1; F/B ratio, Firmicutes-to-Bacteroidetes ratio; LPS, lipopolysaccharide.

A dash (“—”) in the “Pathway affected” column indicates that no specific signaling pathway was reported in the original study.

### 4.1 Regulation of glucose metabolism

Multiple lines of evidence establish that natural polysaccharides consistently upregulate key nodes of the PI3K/Akt/GLUT4 signaling axis. Polysaccharides derived from *Astragalus mongholicus*, *Momordica charantia*, Coix seed, *Gynura divaricata*, and selenium-enriched *Cordyceps militaris* all regulate the expression levels of key proteins in this signaling pathway (13, 125–128) (Table 1). Notably, *Momordica charantia* polysaccharides additionally engage AMPK signaling (13), and Coix seed polysaccharides act through SCFAs-mediated IGF1/PI3K/Akt activation (126), collectively validating PI3K/Akt activation as a conserved anti-diabetic mechanism across taxonomically diverse polysaccharide sources.

The liver is also one of the most important organs to maintain glucose homeostasis, where the equilibrium between gluconeogenesis and glycogen synthesis is vital to glycemic control (129). Natural polysaccharides affect hepatic glucose metabolism via regulating related metabolic enzymes and signaling molecules. Dendrobium officinale polysaccharides regulate blood glucose levels through modulating the glucagon-mediated cAMP-PKA and Akt/FoxO1 pathways, inhibiting hepatic glycogenolysis and gluconeogenesis (130). *Astragalus mongholicus* polysaccharides can also block the process of glycogenolysis and gluconeogenesis to relieve the disorder in glucose metabolism, thus playing a role in regulating metabolism (131).

The other main digestive enzymes for carbohydrates are amylases and glucosidases; the inhibition of these two classes of enzymes delays the uptake of glucose into the bloodstream and can decrease postprandial hyperglycemia (132, 133). Polysaccharides are naturally occurring compounds that have received a lot of attention as possible enzyme inhibitors. Several polysaccharides inhibit either *α*-glucosidase or α-amylase (134). Two polysaccharides extracted and purified from *Rehmannia glutinosa* can inhibit alpha-glucosidase activity as well as alpha-amylase activity, exerting a hypoglycemic effect (135). Polysaccharides from *Chaenomeles speciosa* seeds have α-amylase and α-glucosidase inhibition activities, which are able to delay the hyperglycemic effect after a meal, as well as be involved in regulating blood glucose levels (136).

### 4.2 Protection of pancreatic β-cells

Pancreatic β-cell apoptosis represents a critical event in T2DM pathological progression (137). Natural polysaccharides protect β-cells from injury through regulation of apoptosis-related proteins. *Opuntia milpa alta* polysaccharides attenuate β-cell apoptosis and alleviate T2DM by increasing B-cell lymphoma/leukemia-2 (Bcl-2) expression, reducing Bcl-2-associated X protein (Bax) expression, and decreasing caspase-3 and caspase-9 activities (138). *Lentinus edodes* mycelia polysaccharides alleviate β-cell apoptosis and exert β-cell protective effects through modulating Bax, Bcl-2, cleaved caspase-3, and caspase-1 expression (139).

Natural polysaccharides not only protect β-cells from damage but also promote their insulin secretory function (140). Rose bee pollen polysaccharides promote β-cell proliferation and insulin secretion, maintaining islet function and quality, demonstrating tremendous potential in diabetes treatment (141). Squash polysaccharides improve insulin secretion function in STZ-induced MIN6 cells, exerting protective effects on diabetic models (142).

### 4.3 Anti-inflammatory actions

Chronic low-grade inflammation constitutes an important component of T2DM pathogenic mechanisms (143). The nuclear factor-κB (NF-κB) signaling pathway plays a central regulatory role in inflammatory responses (144), with anti-inflammatory effects achievable through pathway inhibition. *Momordica charantia* polysaccharides reduce NF-κB and caspase-3 expression, exerting anti-inflammatory and anti-apoptotic capabilities and playing important roles in diabetes and diabetic retinopathy treatment (145). Combined inulin and *Lycium barbarum* polysaccharide therapy reduces levels of pro-inflammatory cytokines including NF-κB and NLRP3, improving diabetic inflammatory states (146). Natural polysaccharides regulate the balance between pro-inflammatory and anti-inflammatory cytokines through multiple pathways (147). *Gynostemma pentaphyllum* polysaccharides exert blood glucose regulatory effects by upregulating anti-inflammatory factors IL-4 and IL-10 while downregulating pro-inflammatory factors TNF-*α* and IL-6 (148). *Pseudostellaria heterophylla* polysaccharide PF40 suppresses inflammatory cytokine TNF-α levels while promoting anti-inflammatory cytokine IL-10 levels, exerting hypoglycemic effects (149).

### 4.4 Mitigation of oxidative stress

Oxidative stress plays a significant role in T2DM development and progression, with enhancement of the body’s antioxidant defense system to combat oxidative damage being crucial for T2DM treatment (150). Panax notoginseng leaves polysaccharides increase CAT, SOD, and GSH-Px activities while reducing MDA content, thereby enhancing antioxidant capacity and regulating glucose metabolic disorders (151). *Astragalus mongholicus* polysaccharides reduce MDA levels, elevate GSH, GSH-Px, and SOD activities, alleviate oxidative stress states, and ameliorate renal injury in T2DM rats (152). Nrf2 serves as the master switch for cellular antioxidant responses. Under oxidative stress conditions, Nrf2 translocates from the cytoplasm to the nucleus and binds to antioxidant response elements (ARE), initiating transcription of downstream antioxidant enzyme genes (153, 154). *Lentinus edodes* polysaccharides regulate oxidative stress responses and exert blood glucose regulatory effects in diabetic mice through Nrf2/HO-1 signaling pathway activation (155). Ganoderma applanatum polysaccharides ameliorate insulin resistance and reduce T2DM-induced hepatic and colonic tissue damage through modulating the Nrf2/Keap1-TLR4/NF-κB-Bax/Bcl-2 signaling pathway and suppressing oxidative stress (156).

### 4.5 Regulation of gut microbiota

**Figure 4:**
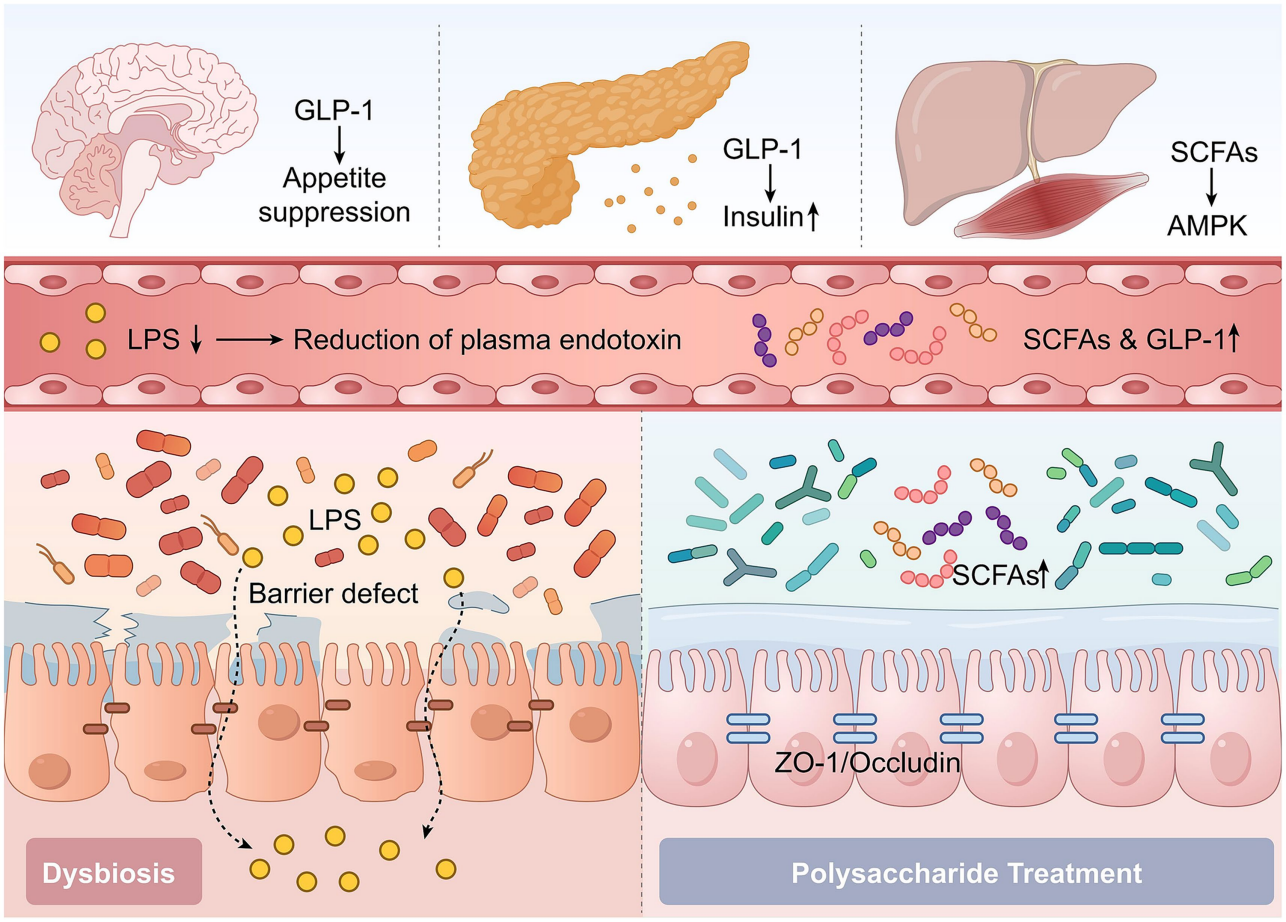
Regulation of gut-metabolic axis by natural polysaccharides. Polysaccharides are used as prebiotic for reversing the gut dysbiosis, increase the abundance of SCFAs producing bacteria and also upregulate tight junction protein (ZO-1, occludin) to restore the intestinal barrier; therefore reduce system endotoxemia (LPS translocation), increase circulating SCFAs & GLP-1, that operate at a distance from their site of origin in distal organs such as the liver, pancreas or brain to promote whole-body metabolic health.

Gut microbiota dysbiosis represents one of the important factors in T2DM pathogenesis (157, 158). As shown in Figure 4, natural polysaccharides, functioning as prebiotics, can selectively promote beneficial bacteria growth and inhibit harmful bacteria proliferation, thereby improving gut microbiota structure (159). *Astragalus mongholicus* polysaccharides restore the normal Firmicutes/Bacteroidetes (F/B) ratio and reduce Clostridia and Proteobacteria abundance (125). In T2DM mice, Astragalus polysaccharides strongly suppressed the potential intestinal pathogen Shigella while promoting beneficial bacteria Allobaculum and Lactobacillus growth (14). *Dendrobium officinale* polysaccharides significantly inhibited harmful *Helicobacter pylori* while promoting probiotic Allobaculum, Bifidobacterium, and Lactobacillus proliferation (15). Following *Cyclocarya paliurus* polysaccharide treatment, gut microbiota structure in T2DM rat models underwent significant alterations, promoting SCFAs-producing bacteria growth (16).

SCFAs are also products of the fermentation process by gut microflora, which are mainly responsible for the degradation of polysaccharides and production of various metabolites (78), playing important roles in the prevention and treatment of T2DM (160, 161). SCFAs can affect the host’s energy metabolism in various ways, such as activating G protein-coupled receptors (162). Polygonati rhizoma polysaccharide promotes SCFAs production and regulates lipid metabolism in T2DM (163). *Ruditapes philippinarum* polysaccharide regulates the intestinal flora, increases SCFAs levels, and improves hyperglycemia (122).

Intestinal barrier dysfunction demonstrates close association with T2DM, leading to increased intestinal permeability and endotoxemia, subsequently triggering systemic inflammation and insulin resistance (164, 165). Natural polysaccharides ameliorate T2DM through restoration of intestinal barrier function. Fructus mori polysaccharides increase tight junction protein expression including claudin-1, occludin, and zonula occludens-1 (ZO-1), repair intestinal barriers, and alleviate diabetic symptoms (166). Polygonatum kingianum polysaccharides improve intestinal barrier integrity and reduce endotoxemia and inflammatory responses through TLR4/NF-κB immune response inhibition, thereby ameliorating glucose and lipid metabolic disorders (167).

**Figure 5:**
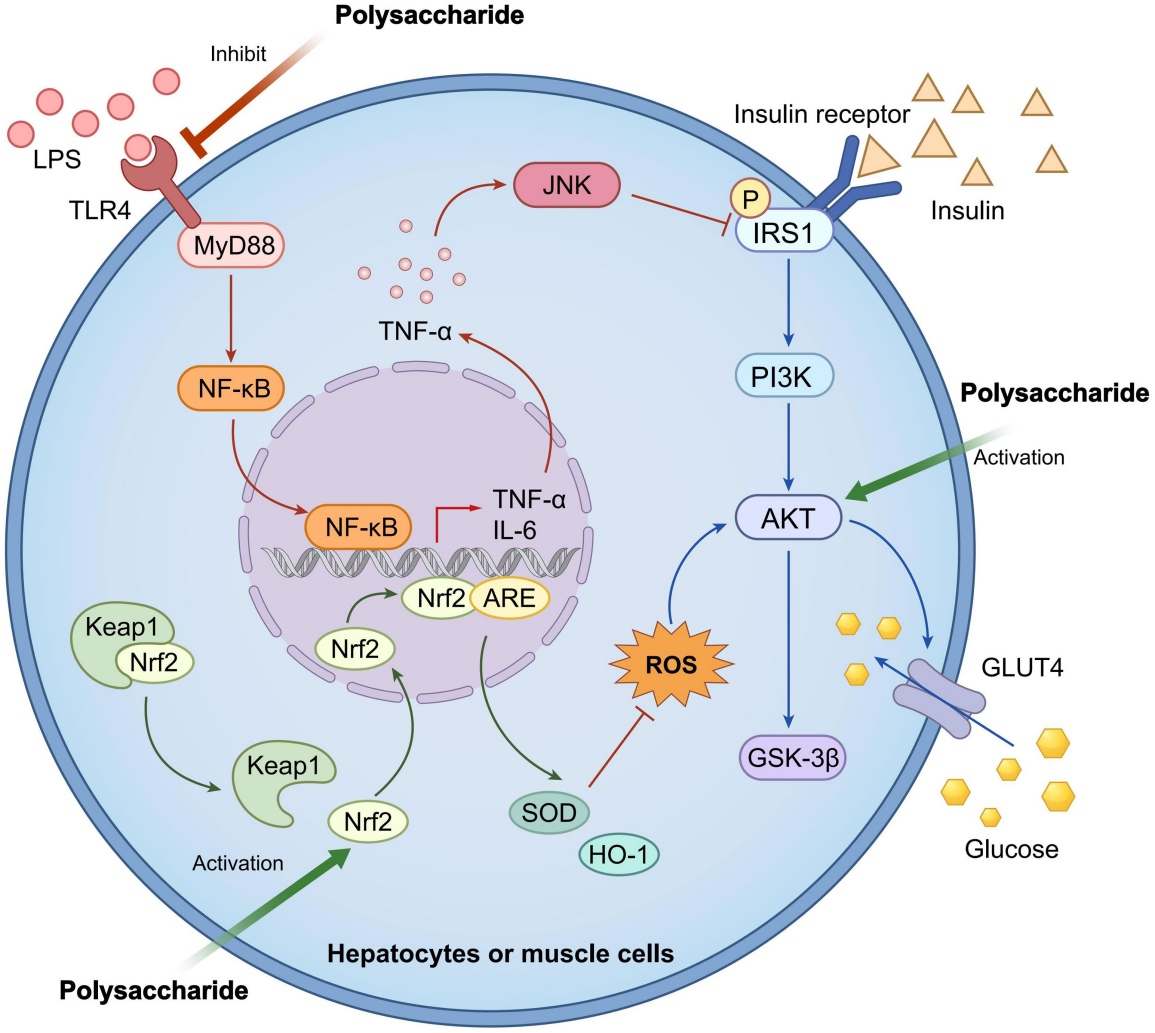
Intracellular signaling crosstalk modulated by natural polysaccharides in T2DM. Polysaccharides restore glucose homeostasis via modulating the cross-talk of insulin signaling, inflammation, and oxidative stress. They activate the PI3K/Akt pathway in order to stimulate GLUT4 translocation. At the same time, they block the TLR4/NF-κB inflammatory axis, reducing cytokine induced serine phosphorylation of IRS-1. Secondly, induction of the Nrf2/ARE antioxidant system scavenge ROS to prevent ROS-mediated suppression of the insulin signal, thus breaking the vicious cycle of IR.

Multi-target and multi-pathway mechanisms are the ways that natural polysaccharides improve T2DM. Here in Figure 5, we summarized the intracellular crosstalk of insulin signaling with oxidative stress and inflammation, which are the pathways that many polysaccharides have been used to treat T2DM. For regulating glucose metabolism, natural polysaccharides activate the IRS/PI3K/Akt signaling pathway, promote GLUT4 expression and translocation to the cell membrane, regulate the hepatic glycogenesis process, and suppress digestive enzyme activity. For protecting pancreatic β-cells, natural polysaccharides can protect β-cells through anti-apoptotic effects and by stimulating insulin release. For anti-inflammatory effects, natural polysaccharides alleviate chronic inflammation by inhibiting related signal pathways of NF-κB and regulating levels of inflammatory factors; in terms of antioxidative effects, natural polysaccharides inhibit oxidative injury by promoting the expression of antioxidant enzymes or activating the Nrf2 signaling pathway. For regulating intestinal microbiota, natural polysaccharides could also improve metabolic health through enhancing microbiota structure, promoting SCFAs production, and restoring intestinal barrier function. These mechanisms are related and synergistic to each other, together forming a molecular basis for natural polysaccharide-mediated T2DM amelioration, providing solid scientific support for using them to prevent or treat T2DM.

## 5 Structure–activity relationship studies of natural polysaccharides

The bioactivity of natural polysaccharides is fundamentally inseparable from their structural characteristics. Even polysaccharides derived from the same botanical source may exhibit markedly divergent biological activities owing to differences in extraction conditions and structural parameters. As discussed in preceding sections, polysaccharides span a wide range of structural complexity—from linear homopolysaccharides to highly branched heteropolysaccharides—and this structural diversity directly underlies the variability in their hypoglycemic efficacy. The following sections systematically examine how individual structural features, including molecular weight, monosaccharide composition, glycosidic linkage types, degree of branching, three-dimensional conformation, and chemical modifications, govern the anti-diabetic activity of natural polysaccharides (see Table 2).

**Table 2:** Summary of key structural parameters governing the hypoglycemic bioactivity of natural polysaccharides, highlighting optimal structural features, and representative examples from the literature.

Structural parameter	Optimal feature for hypoglycemic activity	Representative polysaccharide
Molecular weight	Low molecular weight	*Dendrobium officinale leaves polysaccharides*
Monosaccharide composition	High galactose content (often co-occurring with glucose or galacturonic acid)	*Siraitia grosvenorii* polysaccharide SGP-1-1; red clover polysaccharide RCP-1.1
Glycosidic linkage	β-glycosidic linkages	Green bamboo shoot shell polysaccharide BSSP2a
Degree of branching	Moderate branching	*Cordyceps sinensis* hyperbranched polysaccharides
Three-dimensional conformation	Triple-helix structure	*Pseudostellaria heterophylla* polysaccharide PF40
Chemical modification — sulfation	Appropriate degree of substitution	Sulfated *Sargassum* polysaccharides
Chemical modification — carboxymethylation	Appropriate degree of substitution	Carboxymethylated *Sarcandra glabra* polysaccharides
Chemical modification — acetylation	Appropriate degree of substitution	acetylated *Lavandula angustifolia* polysaccharides
Chemical modification — phosphorylation	Appropriate degree of substitution	*Lavandula angustifolia* polysaccharides

This table summarizes the structural determinants most frequently associated with the hypoglycemic activity of natural polysaccharides, together with the structural features considered optimal, and representative examples reported in the literature. The parameters cover both intrinsic structural attributes — molecular weight, monosaccharide composition, glycosidic linkage, degree of branching, and three-dimensional conformation — and chemical modifications commonly introduced to modulate bioactivity, including sulfation, carboxymethylation, acetylation, and phosphorylation.

The structural featuresends drawn from the cited literature; optimal values may vary depending on polysaccharide origin, extraction method, and the specific bioassay used.

### 5.1 Monosaccharide composition and proportions

The monosaccharide composition is an important component of polysaccharide structure, and the species, ratios, and sequence orders of various monosaccharide units have a direct relationship to the three-dimensional structure and functions of the polysaccharides themselves (168). Hypoglycemic natural polysaccharides possess varied monosaccharide structures, with commonly occurring monosaccharides such as glucose, galactose, and mannose (169). Studies found that the monosaccharide components of *Cassia obtusifolia* polysaccharides CP-30 and CP-40 are different; compared to CP-30, CP-40 has higher xylose content but lower galactose and mannose contents, where these two have different effects on macrophage functioning (170). Gong et al. (171) found that *Siraitia grosvenorii* polysaccharide SGP-1-1 has good antioxidant activity and blood glucose regulatory activities, possibly due to its high content of galactose and mannose among the monosaccharides. Naturally occurring polysaccharides rich in glucose, galactose, or galacturonic acid possess higher antioxidant activity and show great promise for the treatment of T2DM (172). Red clover polysaccharide RCP-1.1, consisting of glucose, galacturonic acid, arabinose, and galactose, has *α*-glucosidase inhibition activity, exerting hypoglycemic and antioxidant effects (173).

### 5.2 Glycosidic linkage types

Glycosidic bonds are the chemical links that connect monosaccharide units, whose type and position of linkage determine polysaccharide structure and conformation, subsequently affecting biological activities (174). Fan et al. (175) found that millet bran polysaccharide MBP-1 has a non-uniform granular shape with alpha- and beta-configurations, showing potent activities for antioxidant and hypoglycemic properties. Green bamboo shoot shell polysaccharide BSSP2a is a homogeneous highly branched β-pyranose polysaccharide with dose-dependent hypoglycemic action, which showed anti-diabetic potential (176). EP50, EP70, and EP90 of *Exocarpium citri grandis* polysaccharides are members of the family of acidic polysaccharides that contain both esterified and non-esterified uronic acids, pyranose-type sugars, and *α*- or β-configuration glycosidic bonds, having beneficial α-glucosidase inhibition and antioxidant activity as well as roles in the prevention and treatment of diabetes and its complications (177).

### 5.3 Molecular weight and biological activity

MW is also an important structural factor affecting the bioactivities of polysaccharides (178), and polysaccharides with various MW ranges have distinct bioeffects (179); for example, low-MW *Dendrobium officinale* leaves polysaccharides are more effective in reducing hyperglycemia and increasing SCFAs associated with gut bacteria (180). Medium molecular weight polysaccharides may augment the immunogenic activity of mature DCs (181), whereas high molecular weight polysaccharides have anti-inflammatory and immunosuppressive effects (182). However, excessively high MW causes low solubility and high viscosity that limits bioavailability (183). Three yam polysaccharides (HSY) of different MWs—HSY-I, HSY-II, and HSY-III—with differences in hypoglycemic activities. Except for HSY-III, the other two fractions, namely HSY-I and HSY-II, have blood glucose-lowering effects (184). Of the two polysaccharide constituents obtained from hippophae rhamnoide l, PHTP and PHBP, the lower molecular weight PHBP has higher water solubility and better *α*-amylase inhibition activity, which showed stronger hypoglycemic activity (185). Wan et al. (186) showed that the low MW thornless *Rosa roxburghii* polysaccharides had better free radical scavenging ability and α-glucosidase inhibitory activity, possessing high hypoglycemic biological activity.

### 5.4 Branching degree and conformation

The polysaccharide branching degree and spatial conformation are more advanced structural information that affects biological activity, with the two being interrelated and together deciding polysaccharide-receptor binding ability and biological functions (187, 188). The hyperbranched structure of *Cordyceps sinensis* hyperbranched polysaccharides could greatly activate the activity of macrophages (189). Heteropolysaccharide fraction BBP-24-3 isolated from blackberry fruits has excellent α-glucosidase inhibitory activity because of its α-helix and random coil structures, which play an important role in the regulation of blood sugar levels (190). *Pseudostellaria heterophylla* polysaccharide PF40 has a non-crystal triple helix conformation, and its monomer shows a branched chain morphology which extends from the core to the outside, establishing the foundation of its antidiabetic activity (191).

### 5.5 Effects of chemical modifications on activity

Chemical modification is also a promising method to improve the physicochemical properties of polysaccharides as well as enhance their bioactivities through introducing some functional groups. Polysaccharide water solubility, charge distribution, and spatial conformation can be modified to confer new or greatly improved biological activity (192–194).

Sulfation is one of the most commonly used polysaccharide modification techniques. Sulfated *Sargassum* polysaccharides have higher antioxidant activity and hypoglycemic activity, promoting glucose metabolism (195). Three sulfated SPPs (S-SPP1-4, S-SPP1-6, and S-SPP1-8) all exhibited higher hypoglycemic activity than the original SPs, indicating that proper sulfation modification could increase the polysaccharides’ antioxidant activity and hypoglycemic effects (195). Carboxymethylation increases the water solubility and bioactivities by introducing a carboxymethyl group on the chain of polysaccharides (193). Carboxymethylated *Sarcandra glabra* polysaccharides have *α*-glucosidase inhibition activity, which can regulate blood sugar levels (196); while acetylation mainly improves the water solubility of polysaccharides and their hydrophobicity (197). The study by Wu et al. (198) reported that acetylated *Lavandula angustifolia* polysaccharides showed increased antioxidant and blood sugar-regulating activity. The modification of phosphorylation can change the charge properties as well as conformation of the molecule by adding phosphate groups (199). According to Gu et al. (200), the *Lavandula angustifolia* polysaccharides obtained via ultrasound-assisted extraction were modified by phosphorylation for enhancing their antioxidant and antihyperglycemic activities.

The hypoglycemic activities of natural polysaccharides demonstrate close associations with their molecular weight, monosaccharide composition, glycosidic linkage types, branching degree, conformation, and chemical modifications. Comprehensive understanding of these structure–activity relationships provides theoretical foundations for developing novel polysaccharide-based hypoglycemic agents and indicates directions for improving polysaccharide activities through structural optimization.

## 6 Future research perspectives

Natural polysaccharides are promising agents for the management of T2DM but many key issues still need to be addressed scientifically. Research efforts should focus on the following areas: mechanism of action, structure–activity relationship investigation, new technologies adoption, and translation to the clinic.

### 6.1 In-depth mechanistic investigations

#### 6.1.1 Polysaccharide-receptor interaction

Natural polysaccharides exhibit the immunoregulatory and metabolism-regulating effect by interacting with cell surface receptors. Biological macromolecules can bind with pattern recognition receptors including TLR4, binding to activate downstream signaling pathways (201), but the exact locations of these sites as well as their affinities and whether they involve structure-selective recognition are still unclear. Polysaccharide-receptor interaction analyses with advanced biophysics techniques such as surface plasmon resonance should be conducted (202). Crosstalks among the receptor-mediated pathways need to be clarified, especially as it concerns how the TLR4 signal is activated via a MyD88-dependent and TRIF-dependent pathway (203). Understanding selective pathway activation will allow for precise control of biological effect.

#### 6.1.2 Epigenetic regulation

Epigenetic regulation, including DNA methylation and histone modification plays key roles in the development of T2DM (204, 205). These are potential modes by which polysaccharides could induce long-term hypoglycemia (206, 207) and further studies using WGBS, chromatin immunoprecipitation sequencing, and transcriptomics in order to map the overall polysaccharide induced epigenetics landscape (208). It is worth noting that whether polysaccharides could reverse the aberrant epigenetic marks in the process of β-cell dedifferentiation deserves special attention.

#### 6.1.3 Mitochondria function

Mitochondria dysfunction is an underlying pathophysiological mechanism in T2DM (209, 210) and the role of polysaccharides in regulating mitochondria biogenesis via PGC-1α, and their involvement with the regulation of mitochondria via DRP1, MFN2, etc., need to be further explored (211). Real-time tracking and functional analysis would reveal how mitochondria respond during treatment by polysaccharides.

#### 6.1.4 Gut-pancreas axis

Gut-pancreatic regulation is a two-way communication system that includes the secretion of incretins (212), microbial metabolites (213), and others. Polysaccharides may also affect the microbiota, although causality between changes in microbiota and health outcomes will need to be more carefully examined using methods like fecal microbiota transplants (214, 215). Metagenomics, metabolomics need to systematically describe the functional microbiota change (216). Single cell sequencing can be used to determine which sub-populations of cells in the gastrointestinal tract are targeted by polysaccharides (217).

### 6.2 Structure–activity relationship elucidation

The high structural complexity of polysaccharides requires more sophisticated tools for analysis. Tandem mass spectrometry will allow detailed structural characterization at the fine level (218). Nuclear magnetic resonance spectroscopy could be used to identify glycosidic linkages (219). Computer modeling via molecular dynamics should yield predictions on three dimensional structures (220). Spatial conformations could also be determined using atomic force microscope and transmission electron microscope (221).

Polysaccharides can be modified with chemical groups such as sulfation, phosphorylation or carboxymethylation to increase their biological activity (197), but site-directed modification approaches must be developed. Computational rational drug design and QSAR models need to forecast the contribution of each structural parameter to hypoglycemic actions (222, 223). Artificial intelligence can speed up the search for highly active polysaccharides (224). The conformation should be studied as a function of temperature, pH, and ionic strength to understand the effect on polysaccharide structure (225, 226). Structural evolution during *in vivo* GI conditions is also important to provide mechanistic insight into this process (227).

### 6.3 Emerging technology applications

#### 6.3.1 Multi-omics integration

Systems biology approach that integrates genomic, transcriptomic, proteomic and lipidomic data could build a global regulatory network (228). Metabolomics and lipidomics describe the impact of polysaccharides on glucose-lipid metabolism (229, 230), whereas joint analysis of the metagenome and metabolome provides insight into microbiota-metabolites-host interaction (231). Single-cell RNA sequencing identifies the response in specific islet cell subpopulations upon polysaccharide exposure (232), while spatial transcriptomics retains the information on architecture of tissues (233).

#### 6.3.2 Nanodelivery systems

Poor oral bioavailability limits the clinical applications. Nanodelivery systems that contain lipid or polymer nanoparticles can circumvent those barriers (234, 235). Nanocarriers can also be used for stimuli responsive and/or controlled drug delivery (236) or ligand modified, to target the site of action (237).

#### 6.3.3 Artificial intelligence

AI technologies facilitate structure–activity relationship predictive modeling and rapid candidate screening (238). Integration of clinical, genomic, and metabolomic data contributes to patient stratification. Deep learning assists medical imaging, enhances accuracy, performs data analysis, and accelerates drug development (239).

#### 6.3.4 Organoid models

Organoid models offer pathologically relevant platforms (240). Pancreatic islet organoids allow for the evaluation of β-cell function (241), intestinal organoids test gut barrier function and incretin secretion (242, 243) or multi-organ-on-chips model metabolism and inter-organ interactions (244). Patient derived iPS-cells enable personalized medicine studies (245).

### 6.4 Clinical translation

Rigorous RCTs are needed to establish efficacy and safety (11). Biomarker guided adaptive design could help increase the chance of success (246, 247). Polysaccharide combination therapy with metformin, SGLT2 inhibitors and/or GLP-1 agonists should be explored. Synergetic combinations of polysaccharides of distinct origin and/or probiotics show potential for the treatment of IR (248). Still, functional foods will need further refinement of the processing steps as well as validation for their effectiveness over time. Further, customized therapies depending on individual gut flora and metabolism are likely to follow (249–251).

## 7 Conclusion

Natural polysaccharides represent a highly promising class of bioactive macromolecules for the prevention and treatment of T2DM. This review has systematically summarized the multi-target mechanisms through which polysaccharides from plant, fungal, marine, and animal sources alleviate T2DM pathology. These mechanisms include enhancement of insulin sensitivity via the PI3K/Akt and AMPK signaling pathways, preservation of pancreatic β-cell mass and function, suppression of chronic inflammation through NF-κB and NLRP3 pathways, attenuation of oxidative stress via Nrf2/HO-1 activation, and restoration of gut microbiota homeostasis with consequent improvement in intestinal barrier integrity and SCFAs metabolism. Collectively, these findings underscore the multi-dimensional therapeutic value of natural polysaccharides in addressing the complex pathophysiology of T2DM.

Structure–activity relationship analyses reveal that hypoglycemic bioactivity is tightly governed by molecular weight, monosaccharide composition, glycosidic linkage type, degree of branching, three-dimensional conformation, and the presence of chemical modifications. Polysaccharides with low molecular weight, high galactose content, β-glycosidic linkages, moderate branching, triple-helix conformations, and strategic derivatizations (e.g., sulfation, carboxymethylation) generally exhibit superior anti-diabetic activities.

Despite these encouraging findings, several scientific challenges remain before the therapeutic promise of natural polysaccharides can be fully realized. The majority of current mechanistic evidence derives from *in vitro* cell models and rodent studies, and whether the observed pathways — ranging from TLR4/NF-κB modulation to gut microbiota remodeling — operate with comparable relevance in human physiology remains to be rigorously established. Moreover, the structural complexity of polysaccharides often precludes unambiguous structure–activity attribution, making it difficult to pinpoint which specific motifs drive particular biological effects. Future mechanistic research should therefore prioritize the integration of multi-omics profiling, computational structural modeling, and human-relevant platforms such as intestinal organoids and microphysiological systems, which together can bridge the gap between molecular-level observations and whole-organism outcomes.

From the perspective of clinical translation and industrialization, several practical bottlenecks must be addressed. The inherent heterogeneity of polysaccharides — arising from species differences, seasonal variation, geographic origin, and extraction methodology — complicates standardization and quality control, underscoring the need for harmonized preparation and characterization protocols. Oral bioavailability constitutes a second critical bottleneck, as high-molecular-weight polysaccharides are prone to gastrointestinal degradation and exhibit limited intestinal permeability; advanced delivery platforms including nanoparticle encapsulation, hydrogels, and pH-responsive carriers offer promising solutions. Finally, well-designed randomized controlled trials remain scarce, leaving the translation from preclinical efficacy to clinical benefit largely unvalidated. Overcoming these barriers will require sustained interdisciplinary collaboration across food science, pharmaceutical engineering, clinical medicine, and regulatory science, positioning natural polysaccharides as viable functional food ingredients, nutraceuticals, and lead compounds for next-generation anti-diabetic therapeutics.
