# Correction: Molecular mechanisms and structure—activity relationships of natural polysaccharides in ameliorating type 2 diabetes mellitus: a comprehensive review

**DOI:** 10.3389/fnut.2026.1912903

**Published:** 2026-07-08

**Authors:** Frontiers Production Office

**Affiliations:** Frontiers Media SA, Lausanne, Switzerland

**Keywords:** glucose metabolism, insulin resistance, molecular mechanisms, natural polysaccharides, pancreatic β-cells, structure–activity relationships, type 2 diabetes mellitus

The following **References** were removed from the published article, as they were found not to be relevant to the theme of the manuscript. The corresponding in-text reference citations have been removed, and all subsequent reference citation numbers have been updated.

- **Reference 26** — Ali Z, Rafique H, Tahir RA, Saeed T, Rasheed MA, Khan I, et al. Clinical and computational exploration of red date fruit vinegar: synergistic effects on cardiovascular and type 2 diabetes pathways. *Front Nutr*. (2025) 12:1557733. doi: 10.3389/fnut.2025.1557733
- **Reference 63** — Rafique H, Hu X, Ren T, Dong R, Aadil RM, Zou L, Sharif MK, Li L. Characterization and exploration of the neuroprotective potential of oat-protein-derived peptides in PC12 cells and scopolamine-treated zebrafish. *Nutrients*. (2023) 16:117. doi: 10.3390/nu16010117
- **Reference 74** — Dong R, Peng K, Shi L, Niu Q, Rafique H, Liu Y, et al. Oat bran prevents high-fat-diet induced muscular dysfunction, systemic inflammation and oxidative stress through reconstructing gut microbiome and circulating metabolome. *Food Res Int*. (2023) 172:113127. doi: 10.1016/j.foodres.2023.113127
- **Reference 82** — Dong R, Rafique H, Niu Q, Zeng X, Messia MC, Yuan L, et al. Interaction of oat bran and exercise training improved exercise adaptability via alleviating oxidative stress and promoting energy homeostasis. *Food Funct*. (2024) 15:11508–24. doi: 10.1039/d4fo03374d
- **Reference 89** — Rafique H, Dong R, Tianqi L, Ma Z, Hu X, Khalid MZ, et al. Oat peptide ameliorates cognitive impairment via mediating gut-brain axis in mice: a multi-omics approach. *J Agric Food Res*. (2025) 24:102394. doi: 10.1016/j.jafr.2025.102394
- **Reference 101** — Akhtar M, Rafique H, Alam Y, Khalid MZ, Zhang J, Alsulami T, et al. Pectin (RG-1)-like polysaccharides isolated from *Gastrodiae rhizoma* via fractional ethanol precipitation: potent inhibitors of pro-inflammatory enzyme modulation targeting iNOS and COX-2. *Int J Biol Macromol*. (2025) 322:146784. doi: 10.1016/j.ijbiomac.2025.146784

The publication date for Reference 20 has been corrected to 2023: Freeman AM, Acevedo LA, Pennings N. *Insulin Resistance*. Treasure Island, FL: StatPearls Publishing (2023).”

Textual corrections have been made throughout the published article to update statistical facts, refine biological terminology, and remove redundant passages.

A correction has been made to **Section 1**, Paragraph 1:

The incorrect sentence reads: “In 2021, the number of people with diabetes was as high as 529 million worldwide, that are expected to increase to 1.31 billion by the year 2050, and T2DM accounts for almost 96% of all such cases, while global health care costs due to diabetes have reached as high as $966 billion in 2021 (1, 2).”.

The sentence has been corrected to “In 2021, the number of people with diabetes was as high as 529 million worldwide, and it is expected to exceed 1.31 billion by 2050. T2DM accounts for almost 96% of all such cases, while global health care costs due to diabetes reached as high as $966 billion in 2021 (1, 2).”.

A correction has been made to the **Section 1**, Paragraph 3:

The incorrect passage reads: “Natural polysaccharides are natural high-molecular-weight carbohydrate macromolecules found ubiquitously in plants, fungi, and seaweeds, and have shown great promise for the prevention/treatment of T2DM because they are readily available with good safety profiles and various biological activities (8, 9).”

The passage has been corrected to “Natural polysaccharides are natural high-molecular-weight carbohydrate macromolecules found ubiquitously in plants, fungi, marine organisms, and animals, and have shown great promise for the prevention/treatment of T2DM because they are readily available with good safety profiles and various biological activities (8, 9).”

A correction has been made to **Section 3**, *Section 3.2*, Paragraph 4.

The incorrect sentence reads: “Polysaccharides from *Inonotus obliquus* possess strong antioxidant, antibacterial, anti-diabetic, anti-obesity, and immune-modulating activity (109).”

The sentence has been corrected to “Polysaccharides from *Inonotus obliquus* possess strong antioxidant, antibacterial, and immune-modulating activity (103).”

A correction has been made to **Section 4**, *Section 4.1*, Paragraph 1.

The incorrect passage reads: “Polysaccharides derived from *Astragalus mongholicus, Momordica charantia*, Coix seed, *Gynura divaricata*, and selenium-enriched *Cordyceps militaris* all elevate p-PI3K, p-Akt, and membrane-associated GLUT4 in HFD/STZ-induced rodent models and insulin-resistant cell lines (13, 131–134) (https://www.frontiersin.org/journals/nutrition/articles/10.3389/fnut.2026.1810613/preview/1810613-xml/2#tab1Table 1).”

**Table 1:** Molecular mechanisms by which natural polysaccharides ameliorate type 2 diabetes mellitus.

Agents	Source	Model	Key molecular changes	Pathway affected	Target mechanism	References
*Astragalus mongholicus* polysaccharides	*Astragalus mongholicus*	HSHFD and STZ-induced SD rats	PI3K↑, Akt↑, GLUT4↑	PI3K/Akt	Enhancement of insulin signaling pathways	(125)
*Momordica charantia* l. polysaccharides	*Momordica charantia* l.	HFD and STZ-induced C57BL/6 mice	p-IRS1/IRS1↓, p-PI3K/PI3K↑, p-Akt/Akt↑, GLUT4↑, p-GSK-3β↑, FoxO1↓, p-AMPK/AMPK↑, PPARα↑, PGC1α↑, SIRT1↑	IRS1/PI3K/Akt, AMPK	Enhancement of insulin signaling pathways	(13)
Coix seed polysaccharides	Coix seed	HFD and STZ-induced C57BL/6J mice	IGF1↑, IGF1R↑, IRS↑, PI3K↑, Akt↑	IGF1/PI3K/Akt	Enhancement of insulin signaling pathways	(126)
Selenium-enriched *Cordyceps militaris* polysaccharides	*Cordyceps militaris*	HepG2 cells	p-Akt/Akt↑, GLUT4↑, p-PI3K/PI3K↑	PI3K/Akt	Promotion of peripheral tissue glucose transport	(127)
*Gynura divaricata* polysaccharides	*Gynura divaricata*	HSHFD and STZ-induced C57BL/6 mice	p-PI3K↑, p-Akt↑, GLUT-4↑	PI3K/Akt/GLUT-4	Promotion of peripheral tissue glucose transport	(128)
*Dendrobium officinale* polysaccharides	*Dendrobium officinale*	HFD and STZ-induced C57BL/6J mice	p-Akt/Akt↑, p-FoxO1/FoxO1↑, G6Pase↓, PEPCK↓, PKA-C↓, p-PKA↓	cAMP-PKA, Akt/FoxO1	Modulation of hepatic glucose metabolism	(130)
*Astragalus mongholicus* polysaccharides	*Astragalus mongholicus*	Transport-induced 1-day-old chicks	PGC-1α↑, SIRT1↓, p-AMPK/AMPK↑, PPARγ↓	PGC-1α/SIRT1/AMPK/PPARα/PPARγ	Modulation of hepatic glucose metabolism	(131)
*Rehmannia glutinosa* polysaccharides	*Rehmannia glutinosa*	STC-1 cells	PI3K↑, p-Akt↑	PI3K/Akt	Inhibition of digestive enzyme activities	(135)
*Opuntia milpa alta* polysaccharides	*Opuntia milpa alta*	INS-1 cells	Bcl-2↑, Bax↓, caspase-3↓, caspase-9↓, cleaved caspase-3↓, cleaved PARP↓	Nrf2	Anti-apoptotic effects	(138)
*Lentinus edodes mycelia* polysaccharides	*Lentinus edodes mycelia*	INS-1 cells	Bax/Bcl-2↓, cleaved caspase-3↓, cleaved caspase-1↓, p-p38/p38↓, p-JNK/JNK↓, nucleus p65↓, nucleus Nrf2↑	p38 MAPK, JNK, NF-κB, Nrf2	Anti-apoptotic effects	(139)
Bee pollen polysaccharide from *Rosa rugosa* thunb. (*Rosaceae*)	*Rosa rugosa* thunb. (*Rosaceae*)	MIN6 cells, alloxan-induced C57BL/6J mice	p-p38/p38↑, p-Erk/Erk↑, p-Akt/Akt↑	Akt, MAPK	Enhancement of insulin secretion	(141)
Squash polysaccharides	Squash	MIN6 cells	ATP↑, Ca^2+^↑, caspase-3↓	—	Enhancement of insulin secretion	(142)
*Momordica charantia* polysaccharide	*Momordica charantia*	STZ-induced SD rats	NF-κB↓, caspase-3↓	NF-κB	Suppression of NF-κB signaling pathway	(145)
*Lycium barbarum* polysaccharide	*Lycium barbarum*	HFD and STZ-induced SD rats	MCP-1↓, IL-18↓, NF-κB↓, NLRP3↓, FGFR4↓, FXR↑, FGF15↓	FXR-FGF15-FGFR4, NF-κB	Suppression of NF-κB signaling pathway	(146)
*Gynostemma pentaphyllum* herb. polysaccharides	*Gynostemma pentaphyllum* herb.	STZ-induced Kunming mice	IL-4↑, IL-10↑, TNF-α↓, IL-6↓	—	Modulation of inflammatory factor balance	(148)
*Pseudostellaria heterophylla* polysaccharides	*Pseudostellaria heterophylla*	HFD and STZ-induced Wistar rats	IL-10↑, TNF-α↓	—	Modulation of inflammatory factor balance	(149)
*Panax notoginseng* leaves polysaccharides	*Panax notoginseng* leaves	HepG2 cells	CAT↑, SOD↑, GSH-Px↑, MDA↓	—	Enhancement of antioxidant enzyme activities	(151)
*Astragalus mongholicus* polysaccharides	*Astragalus mongholicus*	HSHFD and STZ-induced SD rats	GSH↑, GSH-Px↑, SOD↑, MDA↓	—	Enhancement of antioxidant enzyme activities	(152)
*Lentinus edodes* polysaccharides	*Lentinus edodes*	HSHFD and STZ-induced Kunming mice	MDA↓, SOD↑, GSH↑, Nrf2↑, HO-1↑	Nrf2/HO-1	Activation of Nrf2 pathway	(155)
*Ganoderma applanatum* polysaccharides	*Ganoderma applanatum*	HSHFD and STZ-induced Kunming strain mice	Bax↓, Caspase-3↓, Bcl-2↑, TLR4↓, MyD88↓, p-NF-κB P65/NF-κB P65↓, SOD↑, CAT↑, GSH-Px↑, Nrf2↑, HO-1↑, Keap1↓	Nrf2/Keap1-TLR4/NF-κB-Bax/Bcl-2	Activation of Nrf2 pathway	(156)
*Astragalus mongholicus* polysaccharides	*Astragalus mongholicus*	HSHFD and STZ-induced SD rats	Restore Firmicutes/Bacteroidetes (F/B) ratio, clostridia↓, proteobacteria↓, PI3K↑, Akt↑, GLUT4↑, TLR4↓, p-IκB/IκB↓, p-NF-κB/NF-κB↓	PI3K/Akt, TLR4/NF-kB	Improvement of microbiota structure	(125)
*Astragalus mongholicus* polysaccharides	*Astragalus mongholicus*	HFD and STZ-induced C57BL/6J mice	Shigella↓, Allobaculum↑, Lactobacillus↑	—	Improvement of microbiota structure	(14)
*Dendrobium officinale* polysaccharide	*Dendrobium officinale*	HFD and STZ-induced C57BL/6J mice	*Helicobacter*↓, *Allobaculum*↑, *Bifidobacterium*↑, Lactobacillus↑, TLR4↓, TRAM↓, TRIF↓, p-IKKβ↓, p-p65↓	LPS/TLR4/TRIF/NF-kB	Improvement of microbiota structure	(15)
*Cyclocarya paliurus* polysaccharides	*Cyclocarya paliurus*	HFD and STZ-induced SD rats	SCFAs-producing bacteria↑, SCFAs↑, GPR41↑, GPR43↑, GPR109a↑	—	Improvement of microbiota structure	(16)
*Polygonati rhizoma* polysaccharide	*Polygonati rhizoma*	-	SCFAs↑, GPCR41↑, GPCR43↑	PI3K/Akt, TLR4/MyD88/NF-κB	Promotion of SCFAs generation	(163)
*Ruditapes philippinarum* polysaccharides	*Ruditapes philippinarum*	HSHFD and STZ-induced ICR mice	SCFAs↑	—	Promotion of SCFAs generation	(122)
Fructus mori polysaccharides	Fructus mori	HFD and STZ-induced C57BL/6J mice	Claudin-1↑, Occludin↑, ZO-1↑, TLR4↓, MyD88↓, p-IKKβ↓, p-p65↓	TLR4/MyD88/NF-κB	Restoration of intestinal barrier function	(166)
*Polygonatum kingianum* polysaccharides	*Polygonatum kingianum*	HFD-induced SD rats	Occludin↑, ZO-1↑, TLR4↓, IκB-α↑	TLR4/NF-κB	Restoration of intestinal barrier function	(167)

This table summarizes representative natural polysaccharides reported to alleviate T2DM, together with their botanical/biological sources, experimental models, key molecular changes, signaling pathways involved, and the proposed target mechanisms.

Arrows denote the direction of change relative to the diabetic/model control: ↑, up-regulated or increased; ↓, down-regulated or decreased. The prefix “*p*-” indicates the phosphorylated form of a protein; “p-X/X” denotes the ratio of the phosphorylated form to the total protein.

*Animal/cell models:* HFD, high-fat diet; HSHFD, high-sugar high-fat diet; STZ, streptozotocin; SD, Sprague–Dawley; ICR, Institute of Cancer Research; HepG2, human hepatoma cell line; INS-1 and MIN6, rat/mouse pancreatic β-cell lines; STC-1, murine enteroendocrine cell line.

*Molecules and pathways:* PI3K, phosphoinositide 3-kinase; Akt, protein kinase B; IRS1, insulin receptor substrate 1; IGF1/IGF1R, insulin-like growth factor 1/receptor; GLUT4, glucose transporter 4; GSK-3β, glycogen synthase kinase 3β; FoxO1, forkhead box O1; AMPK, AMP-activated protein kinase; PPARα/γ, peroxisome proliferator-activated receptor α/γ; PGC-1α, PPARγ coactivator 1α; SIRT1, sirtuin 1; G6Pase, glucose-6-phosphatase; PEPCK, phosphoenolpyruvate carboxykinase; cAMP, cyclic adenosine monophosphate; PKA, protein kinase A; Bcl-2/Bax, B-cell lymphoma 2/Bcl-2-associated X; PARP, poly(ADP-ribose) polymerase; MAPK, mitogen-activated protein kinase; JNK, c-Jun N-terminal kinase; Erk1/2, extracellular signal-regulated kinase 1/2; NF-κB, nuclear factor κB; IκB/IKKβ, inhibitor of κB/IκB kinase β; TLR4, Toll-like receptor 4; MyD88, myeloid differentiation primary response 88; TRAM/TRIF, TRIF-related adaptor molecule/TIR-domain-containing adapter-inducing interferon-β; NLRP3, NLR family pyrin domain containing 3; FXR, farnesoid X receptor; FGF15/FGFR4, fibroblast growth factor 15/receptor 4; MCP-1, monocyte chemoattractant protein 1; IL-4/6/10/18, interleukin-4/6/10/18; TNF-α, tumor necrosis factor α; CAT, catalase; SOD, superoxide dismutase; GSH, glutathione; GSH-Px, glutathione peroxidase; MDA, malondialdehyde; Nrf2, nuclear factor erythroid 2-related factor 2; HO-1, heme oxygenase 1; Keap1, Kelch-like ECH-associated protein 1; SCFAs, short-chain fatty acids; GPR41/43/109a, G-protein-coupled receptor 41/43/109a; ZO-1, zonula occludens-1; F/B ratio, Firmicutes-to-Bacteroidetes ratio; LPS, lipopolysaccharide.

A dash (“—”)in the “Pathway affected” column indicates that no specific signaling pathway was reported in the original study.

The passage has been corrected to “Polysaccharides derived from *Astragalus mongholicus, Momordica charantia*, Coix seed, *Gynura divaricata*, and selenium-enriched *Cordyceps militaris* all regulate the expression levels of key proteins in this signaling pathway (13, 125–128) (Table 1).”

A correction has been made to **Section 4**, *Section 4.5*, Paragraph 1.

The incorrect passage reads: “In T2DM mice, Astragalus polysaccharides strongly suppressed the potential intestinal pathogen Shigella while promoting beneficial bacteria Alistipes and Lactobacillus growth (14). *Dendrobium officinale* polysaccharides significantly inhibited harmful *Helicobacter pylori* while promoting probiotic Alistipes, Bifidobacterium, and Lactobacillus proliferation (15).”

The passage has been corrected to “In T2DM mice, Astragalus polysaccharides strongly suppressed the potential intestinal pathogen Shigella while promoting beneficial bacteria Allobaculum and Lactobacillus growth (14). *Dendrobium officinale* polysaccharides significantly inhibited harmful *Helicobacter pylori* while promoting probiotic Allobaculum, Bifidobacterium, and Lactobacillus proliferation (15).”

A correction has been made to **Section 5**, *Section 5.1*, Paragraph 1.

The incorrect sentence reads: “Red clover polysaccharide RCP-1.1, consisting of the monomers glucose, galacturonic acid, arabinose, and galactose, has α-glucosidase inhibition activity and antioxidant effect, exerting hypoglycemic and antioxidant effects (179).”

The sentence has been corrected to “Red clover polysaccharide RCP-1.1, consisting of glucose, galacturonic acid, arabinose, and galactose, has α-glucosidase inhibition activity, exerting hypoglycemic and antioxidant effects (173).”

A correction has been made to **Section 5**, *Section 5.4*, Paragraph 1. The incorrect sentence reads:

“Heteropolysaccharide fraction BBP-24-3 isolated from blackberry fruits has excellent α-glucosidase inhibitory activity because of its α-helix and random coil structures, which play an important role in the regulation of blood sugar levels (196).”

The sentence has been corrected to “The heteropolysaccharide fraction BBP-24-3 isolated from blackberry fruits exhibits a uniformly distributed solid spherical structure in 0.1 M NaCl solution and possesses excellent α-glucosidase inhibitory activity (190).”

The following redundant sentence has been removed from **Section 5**, *Section 5.5*, Paragraph 2: “Three sulfated SPPs (S-SPP1-4, S-SPP1-6, and S-SPP1-8) all exhibited higher hypoglycemic activity than the original SPs, indicating that proper sulfation modification could increase the polysaccharides' antioxidant activity and hypoglycemic effects.”

The following text has been removed from the Table 2 footnote: “kDa, kilodalton; ASGPR, asialoglycoprotein receptor; DS, degree of substitution. “β-glucans (fungal)” refers to β-(1 → 3)/(1 → 6)-linked glucans derived from macrofungi.”

The “Key molecular changes” column in Table 1 has been updated across multiple polysaccharide entries to reflect precise protein phosphorylation states, ratios, and updated probiotic genera (e.g., correcting *Alistipes* to *Allobaculum*). Additionally, typographical errors in the “Model” column and abbreviations footnote have been corrected, and the “References” column was renumbered to align with the deleted studies. The corrected Table 1 appears below.

The original version of this article has been updated.

